# COVID-19 Mortality by Race and Ethnicity in US Metropolitan and Nonmetropolitan Areas, March 2020 to February 2022

**DOI:** 10.1001/jamanetworkopen.2023.11098

**Published:** 2023-05-02

**Authors:** Dielle J. Lundberg, Elizabeth Wrigley-Field, Ahyoung Cho, Rafeya Raquib, Elaine O. Nsoesie, Eugenio Paglino, Ruijia Chen, Mathew V. Kiang, Alicia R. Riley, Yea-Hung Chen, Marie-Laure Charpignon, Katherine Hempstead, Samuel H. Preston, Irma T. Elo, M. Maria Glymour, Andrew C. Stokes

**Affiliations:** 1 Department of Global Health, Boston University School of Public Health, Boston, Massachusetts; 2 Department of Health Systems and Population Health, University of Washington School of Public Health, Seattle; 3 Department of Sociology, University of Minnesota, Minneapolis; 4 Minnesota Population Center, University of Minnesota, Minneapolis; 5 Center for Antiracist Research, Boston University, Boston, Massachusetts; 6 Department of Political Science, Boston University, Boston, Massachusetts; 7 Department of Sociology, University of Pennsylvania, Philadelphia; 8 Population Studies Center, University of Pennsylvania, Philadelphia; 9 Department of Epidemiology and Biostatistics, University of California, San Francisco; 10 Department of Epidemiology and Population Health, Stanford University, Stanford, California; 11 Department of Sociology, University of California, Santa Cruz; 12 Institute for Data, Systems, and Society, Massachusetts Institute of Technology, Cambridge; 13 Robert Wood Johnson Foundation, Princeton, New Jersey

## Abstract

**Importance:**

Prior research has established that Hispanic and non-Hispanic Black residents in the US experienced substantially higher COVID-19 mortality rates in 2020 than non-Hispanic White residents owing to structural racism. In 2021, these disparities decreased.

**Objective:**

To assess to what extent national decreases in racial and ethnic disparities in COVID-19 mortality between the initial pandemic wave and subsequent Omicron wave reflect reductions in mortality vs other factors, such as the pandemic’s changing geography.

**Design, Setting, and Participants:**

This cross-sectional study was conducted using data from the US Centers for Disease Control and Prevention for COVID-19 deaths from March 1, 2020, through February 28, 2022, among adults aged 25 years and older residing in the US. Deaths were examined by race and ethnicity across metropolitan and nonmetropolitan areas, and the national decrease in racial and ethnic disparities between initial and Omicron waves was decomposed. Data were analyzed from June 2021 through March 2023.

**Exposures:**

Metropolitan vs nonmetropolitan areas and race and ethnicity.

**Main Outcomes and Measures:**

Age-standardized death rates.

**Results:**

There were death certificates for 977 018 US adults aged 25 years and older (mean [SD] age, 73.6 [14.6] years; 435 943 female [44.6%]; 156 948 Hispanic [16.1%], 140 513 non-Hispanic Black [14.4%], and 629 578 non-Hispanic White [64.4%]) that included a mention of COVID-19. The proportion of COVID-19 deaths among adults residing in nonmetropolitan areas increased from 5944 of 110 526 deaths (5.4%) during the initial wave to a peak of 40 360 of 172 515 deaths (23.4%) during the Delta wave; the proportion was 45 183 of 210 554 deaths (21.5%) during the Omicron wave. The national disparity in age-standardized COVID-19 death rates per 100 000 person-years for non-Hispanic Black compared with non-Hispanic White adults decreased from 339 to 45 deaths from the initial to Omicron wave, or by 293 deaths. After standardizing for age and racial and ethnic differences by metropolitan vs nonmetropolitan residence, increases in death rates among non-Hispanic White adults explained 120 deaths/100 000 person-years of the decrease (40.7%); 58 deaths/100 000 person-years in the decrease (19.6%) were explained by shifts in mortality to nonmetropolitan areas, where a disproportionate share of non-Hispanic White adults reside. The remaining 116 deaths/100 000 person-years in the decrease (39.6%) were explained by decreases in death rates in non-Hispanic Black adults.

**Conclusions and Relevance:**

This study found that most of the national decrease in racial and ethnic disparities in COVID-19 mortality between the initial and Omicron waves was explained by increased mortality among non-Hispanic White adults and changes in the geographic spread of the pandemic. These findings suggest that despite media reports of a decline in disparities, there is a continued need to prioritize racial health equity in the pandemic response.

## Key Points

**Question:**

Why did racial and ethnic disparities in COVID-19 mortality in the US decrease in the Omicron wave compared with the initial wave of the pandemic?

**Findings:**

In this cross-sectional study of 977 018 adults who died from COVID-19, 60.3% of the national decrease in disparities in COVID-19 mortality for non-Hispanic Black compared with non-Hispanic White adults between the initial and Omicron waves could be explained by increases in mortality among non-Hispanic White adults and shifts in mortality to nonmetropolitan areas, where more non-Hispanic White adults reside.

**Meaning:**

This study found that racial and ethnic disparities in COVID-19 mortality decreased nationally for some groups during the first 2 years of the pandemic, but this decrease was mostly explained by increases in mortality for non-Hispanic White adults and changes in pandemic geography.

## Teaser

This cross-sectional study examines contributions to decreases in racial and ethnic disparities in COVID-19 mortality among US adults from the initial wave to Omicron wave.

## Introduction

Considerable research has documented racial and ethnic disparities in mortality during the COVID-19 pandemic in the US.^1,2,3,4,5,6,7,8,9,10,11,12,13^ During 2020, age-standardized death rates from COVID-19 were 2.6 times higher for non-Hispanic American Indian and Alaska Native populations than the non-Hispanic White population.^14^ Other racial and ethnic groups also experienced higher death rates than non-Hispanic White residents; these populations included Hispanic residents (2.3 times higher) and non-Hispanic Black residents (2.1 times higher). Structural racism has been a key driver of national disparities throughout the pandemic.^2,15,16,17,18,19,20,21,22,23,24,25,26,27,28,29,30,31,32,33,34,35^

Prior literature on racial and ethnic disparities in COVID-19 mortality has largely studied the first year of the pandemic. Less is known about how mortality patterns changed during the Delta and Omicron waves in the second year amid increasing vaccination rates. Nationally, racial and ethnic disparities in COVID-19 mortality decreased for some groups between 2020 and 2021.^14^ It remains unknown if these changes were uniform across metropolitan and nonmetropolitan areas. It is also unclear to what extent decreases in disparities reflected reductions in mortality or other factors, such as changes in the geographic spread of the pandemic.

There are multiple reasons to anticipate that pandemic geography and racial and ethnic patterns of COVID-19 mortality may have changed concurrently.^36^ First, the pandemic spread to rural areas in 2021, where a higher proportion of non-Hispanic White people reside.^37,38,39^ Second, differences in vaccine uptake have been reported by race, ethnicity, and urbanicity.^40,41,42,43^

In this study, we examined COVID-19 deaths among adults in the US by race and ethnicity across metropolitan and nonmetropolitan areas from March 1, 2020, to February 28, 2022. Our objective was to understand to what extent national decreases in racial and ethnic disparities in COVID-19 mortality during the first 2 years of the pandemic reflected reductions in mortality vs other factors, such as the pandemic’s changing geography.

## Methods

This cross-sectional study used deidentified, publicly available data and so was exempted from review and informed consent by the Boston University Medical Center Institutional Review Board. The study followed the Strengthening the Reporting of Observational Studies in Epidemiology (STROBE) reporting guideline for cross-sectional studies.

### COVID-19 Mortality Data

We used mortality data from the US Centers for Disease Control and Prevention (CDC) Wide-ranging Online Data for Epidemiologic Research (WONDER) database.^44^ Data were accessed in February 2023. Data for 2020 and 2021 were final, and data for 2022 were provisional. We used *International Statistical Classification of Diseases and Related Health Problems, Tenth Revision* (*ICD-10*) code U07.1 to identify deaths for which COVID-19 was listed anywhere on the death certificate.

Race and ethnicity categories were defined as Hispanic, non-Hispanic American Indian and Alaska Native, non-Hispanic Asian, non-Hispanic Black, non-Hispanic Native Hawaiian and other Pacific Islander, and non-Hispanic White to facilitate comparison with prior CDC estimates.^14^ Race and ethnicity is typically recorded on death certificates by funeral directors who are expected to collect this information from next of kin but may also rely on their own observations.^45^ Prior studies found that the accuracy of race and ethnicity on death certificates was high overall but lower among American Indian and Alaska Native populations.^45,46^

We defined March 1, 2020, through February 28, 2021, and March 1, 2021, through February 28, 2022, as the first and second years of the pandemic, respectively. We divided this 2-year period into the initial wave (March 1 through May 31, 2020), second wave (June 1 through August 31, 2020), Alpha wave (September 1, 2020, through May 31, 2021), Delta wave (June 1 through October 31, 2021), and Omicron wave (November 1, 2021, through February 28, 2022).

Because death counts between 0 and 9 were suppressed, we excluded deaths among individuals aged younger than 25 years given that few deaths occurred at these ages compared with older ages. We aggregated individuals to 3 age groups (25-54, 55-74, and ≥75 years) to minimize suppression.

We condensed the data’s urban and rural classifications into 3 metropolitan and nonmetropolitan categories of residence (large metropolitan, medium and small metropolitan, and nonmetropolitan areas). Large metropolitan areas refer to counties in metropolitan statistical areas with a population of 1 million residents or more. Medium and small metropolitan areas refer to counties in metropolitan statistical areas with a population between 50 000 and 999 999 residents. Nonmetropolitan areas refer to all other counties.^47^

We queried COVID-19 mortality by race and ethnicity across metropolitan and nonmetropolitan categories for each pandemic year, wave, and month. To avoid substantial data suppression, we limited our monthly analyses to the 3 most populous racial and ethnic groups and included non-Hispanic Native Hawaiian and other Pacific Islander adults only in analyses of the pandemic year. Our approach to querying is presented in eAppendix 1 in Supplement 1.

### Population Data

We used April 2020, July 2020, and July 2021 population estimates from the US Census Bureau.^48^ Race and ethnicity in the census is self-reported or reported by a member of the household. We obtained age group–specific monthly population estimates for each race and ethnicity in each metropolitan category by assuming that category-specific populations increased or decreased linearly between estimates and extrapolating for March 2020 and for August 2021 through February 2022.

### Data Imputation

Death counts were imputed when values between 0 and 9 deaths were suppressed. We imputed with random draws from a β distribution with α parameter of 2 and β parameter of 3 and multiplied results by 10. Imputation was limited to nonmetropolitan areas and was done only for the Hispanic population during the first month and non-Hispanic Asian population during the first and second waves.

### Age-Standardized Death Rates

Because racial and ethnic groups in the US have different age distributions, age standardization was necessary.^4,25,49^ We standardized death rates in this study using 3 age groups (25-54, 55-74, and ≥75 years) and used the overall 2020 US population as the standard population. Further details about the age-standardization procedure and our approach to calculating variance are provided in eAppendix 2 in Supplement 1. We estimated age-standardized death rates, changes in rates comparing the initial wave to subsequent waves, rate ratios, and percent changes in rate ratios between waves. All-area rates were calculated as the population-weighted mean of metropolitan-specific rates.

### Statistical Analysis

We decomposed the national change between the initial wave of the pandemic and the Omicron wave in the absolute disparity in age-standardized COVID-19 death rates among non-Hispanic Black compared with non-Hispanic White adults. We also decomposed the national change in disparities for Hispanic compared with non-Hispanic White adults and compared the initial wave with the second, Alpha, and Delta waves. We sought to understand the contribution of the following 4 components to national changes in disparities:

1. The geographically standardized decrease in death rates among non-Hispanic Black or Hispanic adults in a hypothetical population in which the non-Hispanic Black or Hispanic population had the geographic distribution of a standard population.
2. The geographically standardized increase in death rates among non-Hispanic White adults in a hypothetical population in which the non-Hispanic White population had the geographic distribution of a standard population.
3. The change in mortality outcomes associated with shifts in where deaths occurred from metropolitan to nonmetropolitan areas, where more non-Hispanic White adults reside relative to the national geographic distribution (ie, the differential outcomes associated with changes in racial or ethnic–specific and geography-specific mortality rates because of the actual geographic distribution of the US population, with non-Hispanic White adults overrepresented in nonmetropolitan areas).
4. The change in mortality outcomes associated with shifts in the racial and ethnic population composition in metropolitan and nonmetropolitan areas, which we expected to be minor.

The components sum to the national change in disparity. When expressed as percentages, the components sum to 100%. The standard population used for geographic standardization was the aggregate total of Hispanic, non-Hispanic Black, and non-Hispanic White populations, with the mean found across waves. We used the initial wave as the reference wave across all analyses for consistency. Further details about the decomposition analysis are provided in the eAppendix 3 in Supplement 1.

Programming code was developed using R statistical software version 3.6.3 (R Project for Statistical Computing), and replication code is available online on GitHub.^50^ Data were analyzed from June 2021 through March 2023.

## Results

From March 1, 2020, through February 28, 2022, there were death certificates for 977 018 US adults (mean [SD] age, 73.6 [14.6] years; 435 943 female [44.6%]; 156 948 Hispanic [16.1%], 140 513 non-Hispanic Black [14.4%], and 629 578 non-Hispanic White [64.4%]) aged 25 years and older that included a mention of COVID-19. Among 110 526 deaths in the initial wave of the pandemic, 86 263 deaths (78.0%) occurred among adults residing in large metropolitan areas, 18 319 deaths (16.6%) in medium or small metropolitan areas, and 5944 deaths (5.4%) in nonmetropolitan areas (eTable 1 in Supplement 1). In contrast, among 172 515 deaths in the Delta wave, 70 981 deaths (41.1%) were among adults residing in large metropolitan areas. Most deaths were instead among adults living in medium and small metropolitan areas (61 174 deaths [35.5%]) and nonmetropolitan areas (40 360 deaths [23.4%]). Among 210 554 deaths in the Omicron wave, deaths from COVID-19 among adults shifted back to those living in large metropolitan areas (97 716 deaths [46.4%]), while 45 183 deaths (21.5%) occurred among adults in nonmetropolitan areas. Throughout the pandemic, the proportion of the non-Hispanic White population residing in nonmetropolitan areas (25 853 544 of 144 753 329 adults [17.9%]) was 2.8 times larger than that of the Hispanic population (2 304 451 of 36 415 442 adults [6.3%]) and 2.0 times larger than that of the non-Hispanic Black population (2 420 986 of 27 455 724 adults [8.8%]).

### Changes in Mortality Across Metropolitan and Nonmetropolitan Areas

Across the country, death rates decreased for Hispanic (−34.0% [95% CI, −35.8 to −32.3%]), non-Hispanic Asian (−71.2% [95% CI, −74.0 to −68.4%]), and non-Hispanic Black (−49.3% [95% CI, −50.7 to −48.0%]) adults between initial and Delta waves (Table 1). These changes were explained by decreases in mortality in large metropolitan areas (Hispanic: −55.1% [95% CI, −56.9% to −53.3%]; non-Hispanic Asian: −79.3% [95% CI, −82.2% to −76.5%]; non-Hispanic Black: −65.6% [95% CI, −67.1% to −64.2%]). Meanwhile, death rates for these groups increased in medium and small metropolitan and nonmetropolitan areas during this period. At the national level, death rates increased for non-Hispanic American Indian and Alaska Native (36.4% [95% CI, 26.9% to 45.9%]) and non-Hispanic White (22.7% [95% CI, 21.6% to 23.8%]) adults between initial and Delta waves. For non-Hispanic American Indian and Alaska Native adults, increases occurred across metropolitan and nonmetropolitan areas. For non-Hispanic White adults, the increase was explained by nonmetropolitan areas (465.1% [95% CI, 458.1% to 472.0%]) and medium and small metropolitan areas (124.4% [95% CI, 121.6% to 127.1%]). Similar patterns were observed when comparing initial and Omicron waves.

**Table 1.** Changes in Adult COVID-19 Death Rates From the Initial to Delta Wave

Area category	Race and ethnicity	ASDR, No./100 000 PYs^a^		ASDR change from initial to Delta wave, % (95% CI)	RR (95% CI)		RR change from initial to Delta wave, % (95% CI)
		Initial wave	Delta wave		Initial wave	Delta wave	
Large metropolitan	Hispanic	403.0	180.9	−55.1 (−56.9 to −53.3)	1.9 (1.9 to 2.0)	1.5 (1.4 to 1.5)	−23.5 (−25.6 to −21.4)
	Non-Hispanic American Indian and Alaska Native	122.3	193.4	58.1 (32.9 to 83.4)	0.6 (0.5 to 0.7)	1.6 (1.4 to 1.7)	169.4 (111.3 to 227.5)
	Non-Hispanic Asian	240.0	49.6	−79.3 (−82.2 to −76.5)	1.1 (1.1 to 1.2)	0.4 (0.4 to 0.4)	−64.8 (−66.7 to −62.9)
	Non-Hispanic Black	590.7	202.9	−65.6 (−67.1 to −64.2)	2.8 (2.8 to 2.9)	1.7 (1.6 to 1.7)	−41.5 (−43.0 to −40.0)
	Non-Hispanic White	209.3	122.8	−41.3 (−42.4 to −40.2)	1 [Reference]	1 [Reference]	0 [Reference]
Medium and small metropolitan	Hispanic	120.3	240.3	99.7 (92.9 to 106.6)	1.4 (1.3 to 1.5)	1.2 (1.2 to 1.3)	−11.0 (−16.1 to −5.8)
	Non-Hispanic American Indian and Alaska Native	233.1	294.3	26.3 (10.4 to 42.1)	2.7 (2.4 to 3.0)	1.5 (1.4 to 1.6)	−43.7 (−52 to −35.4)
	Non-Hispanic Asian	63.9	99.0	54.9 (39.7 to 70.2)	0.7 (0.7 to 0.8)	0.5 (0.5 to 0.5)	−31.0 (−40.1 to −21.9)
	Non-Hispanic Black	249.2	305.9	22.7 (18.3 to 27.2)	2.9 (2.8 to 3.0)	1.6 (1.5 to 1.6)	−45.3 (−47.8 to −42.8)
	Non-Hispanic White	86.9	195.0	124.4 (121.6 to 127.1)	1 [Reference]	1 [Reference]	0 [Reference]
Nonmetropolitan	Hispanic	83.1	333.9	302.0 (282.4 to 321.7)	1.8 (1.6 to 2.0)	1.3 (1.2 to 1.3)	−28.8 (−37.6 to −20.1)
	Non-Hispanic American Indian and Alaska Native	273.8	374.3	36.7 (23.3 to 50.2)	5.9 (5.3 to 6.5)	1.4 (1.3 to 1.5)	−75.8 (−78.8 to −72.8)
	Non-Hispanic Asian	31.8	107.5	238.4 (169.0 to 307.7)	0.7 (0.4 to 1.0)	0.4 (0.3 to 0.5)	−40.1 (−66.6 to −13.6)
	Non-Hispanic Black	256.4	367.6	43.4 (36.3 to 50.5)	5.5 (5.2 to 5.9)	1.4 (1.3 to 1.4)	−74.6 (−76.4 to −72.8)
	Non-Hispanic White	46.5	262.9	465.1 (458.1 to 472.0)	1 [Reference]	1 [Reference]	0 [Reference]
All	Hispanic	311.9	205.7	−34.0 (−35.8 to −32.3)	2.2 (2.2 to 2.3)	1.2 (1.2 to 1.2)	−46.2 (−47.4 to −45.0)
	Non-Hispanic American Indian and Alaska Native	217.7	297.0	36.4 (26.9 to 45.9)	1.6 (1.4 to 1.7)	1.7 (1.6 to 1.8)	11.2 (1.6 to 20.7)
	Non-Hispanic Asian	205.7	59.2	−71.2 (−74.0 to −68.4)	1.5 (1.4 to 1.5)	0.3 (0.3 to 0.4)	−76.5 (−77.6 to −75.4)
	Non-Hispanic Black	478.5	242.5	−49.3 (−50.7 to −48.0)	3.4 (3.4 to 3.5)	1.4 (1.4 to 1.4)	−58.7 (−59.5 to −57.9)
	Non-Hispanic White	140.0	171.8	22.7 (21.6 to 23.8)	1 [Reference]	1 [Reference]	0 [Reference]

Abbreviations: ASDR, age-standardized death rates; PY, person-year; RR, rate ratio.

^a^ To facilitate comparison across waves of different durations, death rates were annualized and estimates reported in units of COVID-19 deaths per 100 000 person-years.

**Figure 1.**
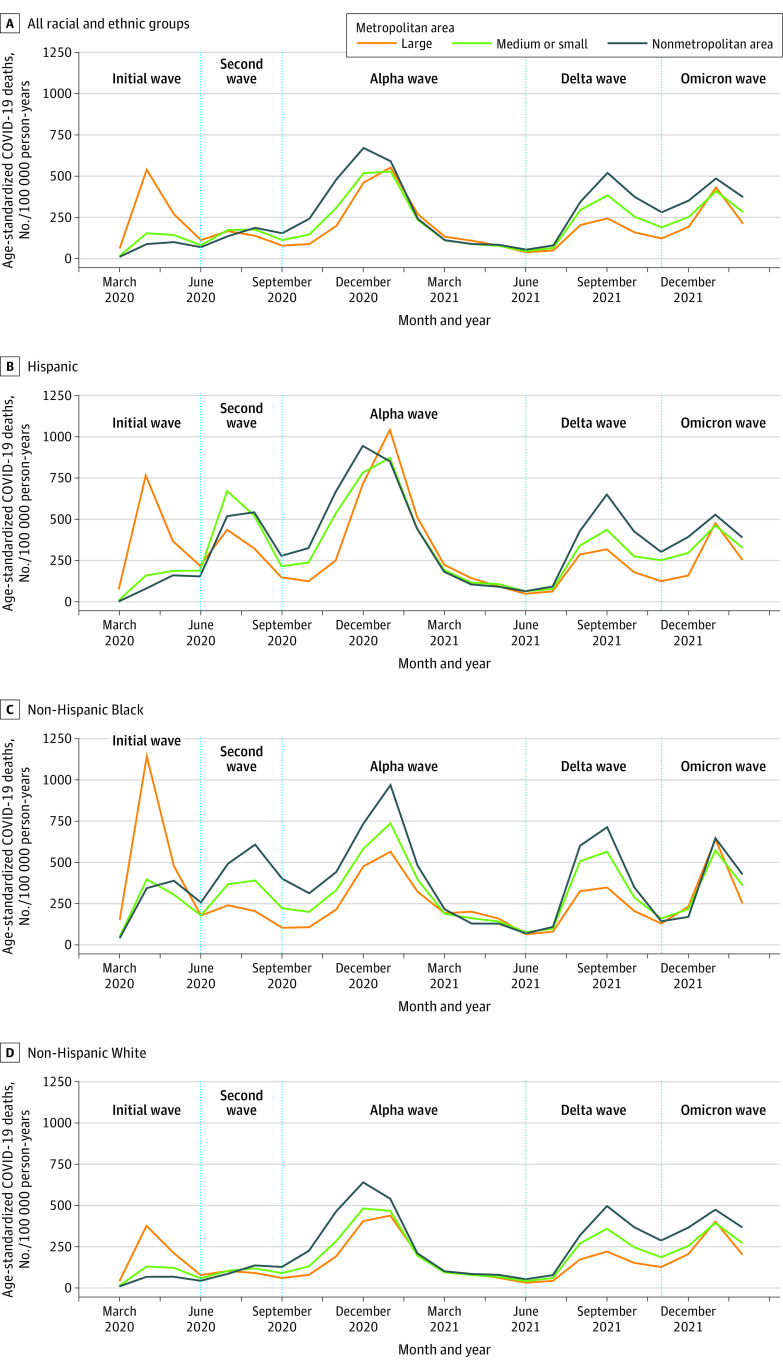
Monthly Adult Age-Standardized COVID-19 Death Rates Death rates were standardized using 3 age groups (25-54, 55-74, and ≥75 years) and the overall US 2020 population as the standard population. Non-Hispanic American Indian and Alaska Native, non-Hispanic Asian, and non-Hispanic Native Hawaiian and other Pacific Islander adults were excluded from this figure due to substantial data suppression at this level of temporal detail. Death rates were annualized and reported in units of COVID-19 deaths per 100 000 person-years.

Figure 1 provides additional temporal detail to understand when mortality shifted to nonmetropolitan areas. Death rates among Hispanic, non-Hispanic Black, and non-Hispanic White adults in nonmetropolitan areas exceeded rates in large metropolitan areas as early as the second wave; for example, among Hispanic adults, rates per 100 000 person-years for nonmetropolitan vs large metropolitan areas were 83 deaths vs 403 deaths in the initial wave and 406 deaths vs 327 deaths in the second wave (eTable 2 in Supplement 1). Death rates among non-Hispanic White adults increased between the initial and Alpha waves (47.7% [95% CI, 46.7%-48.7%]) (eTable 3 in Supplement 1). Death rates for non-Hispanic White adults also increased between the Alpha and Omicron waves, most notably in nonmetropolitan areas (Alpha: 275 deaths/100 000 person-years; Omicron: 375 deaths/100 000 person-years). By the second year of the pandemic, a rural mortality disadvantage was observed for all racial and ethnic groups except non-Hispanic Native Hawaiian and other Pacific Islander adults; for example, the death rate per 100 000 person-years among Hispanic adults in the second year of the pandemic was 307 deaths in nonmetropolitan areas vs 200 deaths in large metropolitan areas and 248 deaths in medium and small metropolitan areas (eTable 4 in Supplement 1). Within nonmetropolitan areas during the second pandemic year, death rates per 100 000 person-years were higher for Hispanic, non-Hispanic American Indian and Alaska Native (374 deaths), and non-Hispanic Black (308 deaths) adults than non-Hispanic White adults (256 deaths) (eTable 4 in Supplement 1).

### Changes in Disparities in COVID-19 Mortality

At the national level, death rates were higher for non-Hispanic Black adults than non-Hispanic White adults during the initial wave of the pandemic (rate ratio [RR], 3.4 [95% CI, 3.4-3.5]) (Table 1). This disparity decreased over time, but death rates among non-Hispanic Black adults were still higher compared with those for non-Hispanic White adults during the Delta (RR, 1.4 [95% CI, 1.4-1.4]) and Omicron (RR, 1.2 [95% CI, 1.1-1.2]) waves. Similarly, the disparity for Hispanic adults compared with non-Hispanic White adults was large in the initial wave (RR, 2.2 [95% CI, 2.2-2.3]) and smaller during the Delta (RR, 1.2 [95% CI, 1.2-1.2]) and Omicron (RR, 1.0 [95% CI, 1.0-1.1]) waves (Table 2). In the initial wave, non-Hispanic Asian adults had higher death rates than non-Hispanic White adults, whereas the Delta and Omicron waves presented the opposite pattern. Disparities remained relatively constant for non-Hispanic American Indian and Alaska Native adults compared with non-Hispanic White adults from the initial wave to the Delta and Omicron waves and for non-Hispanic Native Hawaiian and Other Pacific Islander adults from the first to second pandemic year (eTable 4 in Supplement 1).

**Table 2.** Changes in Adult COVID-19 Death Rates From the Initial to Omicron Wave

Area category	Race and ethnicity	ASDR, No./100 000 PYs^a^		ASDR change from initial to Omicron wave, % (95% CI)	RR (95% CI)		RR change from initial to Omicron wave, % (95% CI)
		Initial wave	Omicron wave		Initial wave	Omicron wave	
Large metropolitan	Hispanic	403.0	255.1	−36.7 (−38.6 to −34.8)	1.9 (1.9 to 2.0)	1.1 (1.1 to 1.1)	−43.4 (−44.8 to −41.9)
	Non-Hispanic American Indian and Alaska Native	122.3	333.4	172.7 (141.2 to 204.1)	0.6 (0.5 to 0.7)	1.4 (1.3 to 1.6)	144.0 (93.1 to 194.9)
	Non-Hispanic Asian	240.0	118.2	−50.8 (−53.9 to −47.6)	1.1 (1.1 to 1.2)	0.5 (0.5 to 0.5)	−55.9 (−57.9 to −54.0)
	Non-Hispanic Black	590.7	311.9	−47.2 (−48.8 to −45.6)	2.8 (2.8 to 2.9)	1.3 (1.3 to 1.4)	−52.7 (−53.9 to −51.6)
	Non-Hispanic White	209.3	233.9	11.7 (10.4 to 13.0)	1 [Reference]	1 [Reference]	0 [Reference]
Medium and small metropolitan	Hispanic	120.3	335.2	178.6 (170.4 to 186.8)	1.4 (1.3 to 1.5)	1.2 (1.2 to 1.2)	−12.6 (−17.6 to −7.6)
	Non-Hispanic American Indian and Alaska Native	233.1	440.5	89.0 (70.6 to 107.5)	2.7 (2.4 to 3.0)	1.6 (1.5 to 1.7)	−40.7 (−49.2 to −32.1)
	Non-Hispanic Asian	63.9	138.0	115.9 (98.3 to 133.6)	0.7 (0.7 to 0.8)	0.5 (0.5 to 0.5)	−32.2 (−41.1 to −23.4)
	Non-Hispanic Black	249.2	326.6	31.1 (26.3 to 35.9)	2.9 (2.8 to 3.0)	1.2 (1.1 to 1.2)	−58.9 (−60.8 to −56.9)
	Non-Hispanic White	86.9	276.9	218.7 (215.4 to 222.0)	1 [Reference]	1 [Reference]	0 [Reference]
Nonmetropolitan	Hispanic	83.1	403.7	386.0 (362.6 to 409.4)	1.8 (1.6 to 2.0)	1.1 (1.0 to 1.1)	−39.7 (−47.1 to −32.2)
	Non-Hispanic American Indian and Alaska Native	273.8	547.6	100.0 (84.3 to 115.7)	5.9 (5.3 to 6.5)	1.5 (1.4 to 1.6)	−75.2 (−78.2 to −72.1)
	Non-Hispanic Asian	31.8	156.0	390.9 (305.2 to 476.5)	0.7 (0.4 to 1.0)	0.4 (0.4 to 0.5)	−39.1 (−65.8 to −12.3)
	Non-Hispanic Black	256.4	345.7	34.8 (27.4 to 42.2)	5.5 (5.2 to 5.9)	0.9 (0.9 to 1.0)	−83.3 (−84.5 to −82.0)
	Non-Hispanic White	46.5	374.7	705.4 (696.7 to 714.1)	1 [Reference]	1 [Reference]	0 [Reference]
All	Hispanic	311.9	285.0	−8.6 (−10.5 to −6.7)	2.2 (2.2 to 2.3)	1.0 (1.0 to 1.1)	−53.2 (−54.2 to −52.2)
	Non-Hispanic American Indian and Alaska Native	217.7	451.9	107.6 (96.3 to 118.8)	1.6 (1.4 to 1.7)	1.7 (1.6 to 1.7)	6.3 (−2.6 to 15.2)
	Non-Hispanic Asian	205.7	122.4	−40.5 (−43.6 to −37.3)	1.5 (1.4 to 1.5)	0.4 (0.4 to 0.5)	−69.5 (−70.7 to −68.3)
	Non-Hispanic Black	478.5	318.4	−33.5 (−34.9 to −32.0)	3.4 (3.4 to 3.5)	1.2 (1.1 to 1.2)	−65.9 (−66.6 to −65.2)
	Non-Hispanic White	140.0	273.4	95.2 (94.0 to 96.5)	1 [Reference]	1 [Reference]	0 [Reference]

Abbreviations: ASDR, age-standardized death rate; PY, person-year; RR, rate ratio.

^a^ To facilitate comparison across waves of different durations, death rates were annualized and estimates reported in units of COVID-19 deaths per 100 000 person-years.

### Decomposition of Changes in Disparities

**Figure 2.**
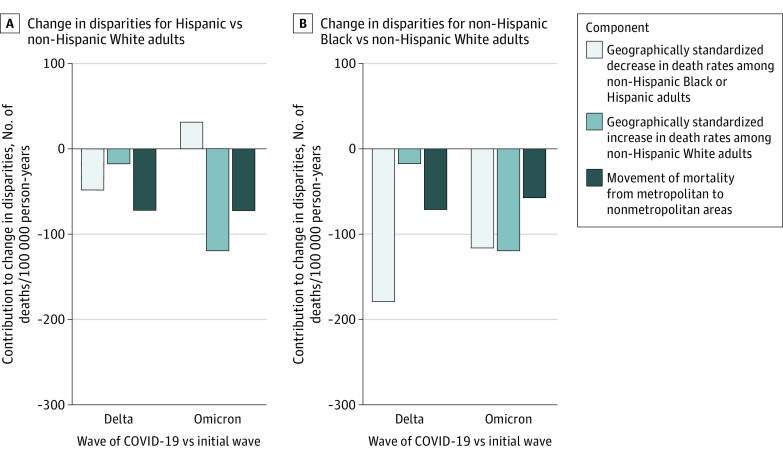
Decomposition of Mechanisms Contributing to National Changes in Racial and Ethnic Disparities For each comparison of racial and ethnic groups and waves, components add up to the total change in disparities. The component consisting of changes in mortality outcomes associated with shifts in the racial and ethnic population composition of metropolitan and nonmetropolitan areas was omitted from the figure because it contributed between −0.2% and 0.1% to the change in disparities across wave and racial and ethnic group comparisons.

We sought to understand mechanisms that may have contributed to national decreases in disparities. The national disparity in age-standardized COVID-19 death rates per 100 000 person-years for non-Hispanic Black compared with non-Hispanic White adults decreased from 339 to 45 deaths, or by 293 deaths, from the initial wave to the Omicron wave (Figure 2). Increases in geographically standardized death rates per 100 000 person-years for non-Hispanic White adults explained 120 deaths (40.7%) in this decrease. Movement of mortality per 100 000 person-years from metropolitan to nonmetropolitan areas explained another 58 deaths (19.6%), and 116 deaths (39.6%) were explained by decreases in geographically standardized death rates among non-Hispanic Black adults. For Hispanic compared with non-Hispanic White adults, the national disparity in age-standardized COVID-19 mortality per 100 000 person-years decreased from 172 to 12 deaths, or by 160 deaths, during this period. This decrease was fully explained by increases in geographically standardized death rates among non-Hispanic White adults (120 deaths/100 000 person-years [74.6%]) and movement of mortality from metropolitan to nonmetropolitan areas (72 deaths/100 000 person-years [45.2%]). Geographically standardized death rates increased for Hispanic adults during this period and thus contributed negatively to the decrease (−32 deaths/100 000 person-years [−19.6%]).

Comparing the Delta wave with the initial wave, increases in geographically standardized death rates for non-Hispanic White adults and movement of mortality from metropolitan to nonmetropolitan areas also explained much of the national decrease in disparities (Figure 2). In the national decrease in disparities in deaths per 100 000 person-years for non-Hispanic Black compared with non-Hispanic White adults from 339 to 71 deaths, or by 268 deaths, these mechanisms contributed to 89 deaths in the decrease (33.1%). In the national decrease in disparities in deaths per 100 000 person-years for Hispanic compared with non-Hispanic White adults from 172 to 34 deaths, or by 138 deaths, the mechanisms contributed to 90 deaths in the decrease (65.0%). Full results of the decomposition, including comparisons of the initial wave with the second and Alpha waves, are presented in eTable 5 in Supplement 1.

## Discussion

In this cross-sectional study of COVID-19 deaths in the US, we found that racial and ethnic disparities for Hispanic and non-Hispanic Black adults compared with non-Hispanic White adults decreased substantially between the initial wave of the pandemic and the Omicron wave. However, most of this reduction in disparities for non-Hispanic Black adults and all of the reduction in disparities for Hispanic adults was explained by increases in geographically standardized mortality among non-Hispanic White adults and geographic shifts of the pandemic to nonmetropolitan areas, where a disproportionate share of non-Hispanic White adults reside.

Our findings contrast with recent media reports suggesting that decreases in racial and ethnic disparities in COVID-19 mortality may represent improvements in public health.^51^ Our work suggests that it may be premature to celebrate reductions in disparities because they did not largely represent reductions in mortality.^13^ In fact, over the study period, death rates increased markedly in all areas for non-Hispanic White and non-Hispanic American Indian and Alaska Native adults and in nonmetropolitan areas for most groups. Within nonmetropolitan areas, death rates were substantially higher for Hispanic, non-Hispanic American Indian and Alaska Native, and non-Hispanic Black adults during the second year of the pandemic than for non-Hispanic White adults.

The emergence of a rural disadvantage in COVID-19 death rates for most racial and ethnic groups during the second year of the pandemic may point to social and structural factors associated with health in rural areas. Rural populations, especially non-Hispanic Black and non-Hispanic American Indian and Alaska Native individuals, have higher rates of chronic diseases, which were associated with increased risk of dying from COVID-19.^52,53^ Health systems that serve rural areas, including the Indian Health Service, have long been underfunded,^54,55^ and Hispanic, non-Hispanic Black, and non-Hispanic American Indian and Alaska Native populations in rural areas report lower access to health care than the non-Hispanic White population. Prior research has further demonstrated the association of declining economic opportunity with negative outcomes in rural health,^56,57^ which was worsened in the COVID-19 pandemic.^58^

This rural disadvantage could also be associated with lower COVID-19 vaccination rates. As of January 2022, 61% of metropolitan residents and 48% of nonmetropolitan residents were fully vaccinated.^59^ This may be partially associated with partisanship. Despite the effectiveness of vaccines in their association with reduced mortality, Republican voters were more vaccine hesitant than other populations and more frequently resided in rural areas.^39,41,60,61^ While vaccination delivery favored older and non-Hispanic White individuals early in the pandemic,^62,63^ the racial and ethnic gap in vaccination rates has since decreased.^64^ However, delivery can still improve.^65,66,67^ As of February 2023, Hispanic, non-Hispanic American Indian and Alaska Native, and non-Hispanic Black adults have received boosters at substantially lower rates than non-Hispanic White adults.^68^ Evidence-based approaches to increasing vaccine and booster uptake include partnerships with faith-based organizations, housing communities, and community organizations; mitigating barriers, such as requirements for photo identification; making vaccines available outside typical working hours; ensuring that clinics are accessible via public transportation; and centering Black and American Indian and Alaska Native officials in outreach efforts.^66,69,70,71,72^ Strategies in rural areas include education of community ambassadors, use of social media, and operation of mobile vaccination sites.^73^ The Panola Project in Alabama is 1 case study of a community leader, Dorothy Oliver, who organized her rural, predominantly Black community to get vaccinated with little government support.^74^ The CDC and health departments could support similar community-led efforts on a wider scale.^75^

Beyond vaccination, additional policy changes could further support racial health equity during the pandemic. Paid sick time and medical leave may ensure that essential workers are able to isolate and recover if they develop COVID-19.^76,77^ Continued rent, eviction, and foreclosure moratoriums and extended unemployment benefits may reduce financial and housing insecurity.^78,79^ Economic reparations, investments in community-based safety, and funding for social programs may address social determinants of health that have been associated with mortality disparities.^28,80,81,82^ Investments in health systems that serve rural areas, including Indigenous health systems, may also help reduce high rural death rates observed for most racial and ethnic groups.^54,55^ Finally, initiatives to diversify the physician workforce and reduce medical racism may contribute to a more equitable pandemic response.^83,84^

### Limitations

This study has several limitations. First, a key limitation of our decomposition analysis was that it was not possible to fully separate racial and ethnic disparities in mortality from geography because residential segregation and other forms of structural racism are associated with where members of racial and ethnic groups live.^15,21,22^ Efforts to geographically standardize COVID-19 mortality estimates early in the pandemic were criticized for decreasing the estimated magnitude of racial and ethnic disparities by controlling for structural racism that made it more likely for Hispanic and non-Hispanic Black individuals to live in urban areas, where COVID-19 death rates were higher.^85^ As our study found, however, death rates in rural areas exceeded those in urban areas as early as the second wave of the pandemic. This suggests that the association of geographic standardization with differences in mortality estimates changed as the pandemic progressed. While geographic standardization remains controversial as a descriptive tool, we believe it is a useful instrument to understand mechanisms associated with disparities in mortality and their changes over time. In this study, such an analysis suggested that the association of structural racism with COVID-19 mortality persists even when changes in the geographic spread of the pandemic may disguise some of its associated outcomes in national mortality statistics.

Second, COVID-19 mortality data for January and February 2022 were provisional, and such mortality data are 75% complete 8 weeks after deaths occurred.^86^ Our data were accessed 11 months after the last death in our study, suggesting that these data may be nearly complete. Third, population estimates for 2022 were not available; some geographic units may have experienced population changes, and we could not account for these changes. Fourth, we used an imputation procedure to replace suppressed data; however, imputation was infrequent. Fifth, owing to constraints related to data suppression, we used 3 broader age categories rather than 10-year categories for age-standardization and did not standardize by sex. Sixth, classification of race and ethnicity on death certificates is less accurate for American Indian and Alaska Native populations.^45,46^ Thus, we may have underestimated mortality for these adults. Seventh, we did not examine death rates among non-Hispanic adults with multiracial backgrounds because prior research has identified significant heterogeneity in this population.^87,88^ Future research should use other data sources to explore important differences that may exist within this population. Eighth, our estimates of COVID-19 mortality may differ from estimates of excess mortality, which include uncounted COVID-19 deaths and deaths indirectly related to the pandemic.^9^ Nonmetropolitan areas and some racial and ethnic groups, such as non-Hispanic Black populations, may have a greater number of uncounted deaths.^9,89^ Understanding how undercounting of COVID-19 deaths varied throughout the pandemic is an important area for future research.

## Conclusions

In this cross-sectional study, we decomposed national decreases in racial and ethnic disparities in age-standardized COVID-19 mortality for Hispanic and non-Hispanic Black adults compared with non-Hispanic White adults over the first 2 years of the pandemic. We found that most of the decrease in disparities for non-Hispanic Black adults and all of the decrease in disparities for Hispanic adults was explained by increases in geographically standardized mortality among non-Hispanic White adults and movement of the pandemic to nonmetropolitan areas rather than reductions in mortality. While advancements toward racial and ethnic health equity have occurred, our study suggests that this work is not finished. Efforts to promote equitable booster distribution, invest in rural health systems, and address structural racism are still urgently needed.

## Notes

Supplement 1. **eTable 1.** Proportion of Population and Adult COVID-19 Deaths by Metropolitan and Nonmetropolitan Area **eTable 2.** Changes in Adult COVID-19 Death Rates From Initial to Second Wave **eTable 3.** Changes in Adult COVID-19 Death Rates From Initial to Alpha Wave **eTable 4.** Changes in Adult COVID-19 Death Rates From First to Second Year of Pandemic **eTable 5.** Full Results for the Demographic Decomposition of the Components Contributing to National Changes in Disparities in COVID-19 Mortality for Hispanic and Non-Hispanic Black Adults vs Non-Hispanic White Adults From Initial to Subsequent Waves **eAppendix 1.** Data-Querying Approach **eAppendix 2.** Age-Standardization Procedure **eAppendix 3.** Decomposition Analysis Derivation and Formulas
eReferences.

Supplement 2.
Data Sharing Statement

## References

[1] Millett GA, Jones AT, Benkeser D, . Assessing differential impacts of COVID-19 on Black communities. Ann Epidemiol. 2020;47:37-44. doi:10.1016/j.annepidem.2020.05.003 32419766 PMC7224670

[2] Chen JT, Krieger N. Revealing the Unequal Burden of COVID-19 by Income, Race/Ethnicity, and Household Crowding: US County Versus Zip Code Analyses. J Public Health Manag Pract. 2021;27(Suppl 1, COVID-19 and Public Health: Looking Back, Moving Forward):S43-S56. doi:10.1097/PHH.0000000000001263 32956299

[3] Mude W, Oguoma VM, Nyanhanda T, Mwanri L, Njue C. Racial disparities in COVID-19 pandemic cases, hospitalisations, and deaths: a systematic review and meta-analysis. J Glob Health. 2021;11:05015. doi:10.7189/jogh.11.05015 34221360 PMC8248751

[4] Luck AN, Preston SH, Elo IT, Stokes AC. The unequal burden of the COVID-19 pandemic: capturing racial/ethnic disparities in US cause-specific mortality. SSM Popul Health. 2022;17:101012. doi:10.1016/j.ssmph.2021.101012 34961843 PMC8697426

[5] Muñoz-Price LS, Nattinger AB, Rivera F, . Racial disparities in incidence and outcomes among patients with COVID-19. JAMA Netw Open. 2020;3(9):e2021892. doi:10.1001/jamanetworkopen.2020.21892 32975575 PMC7519420

[6] Kullar R, Marcelin JR, Swartz TH, . Racial disparity of coronavirus disease 2019 in African American communities. J Infect Dis. 2020;222(6):890-893. doi:10.1093/infdis/jiaa372 32599614 PMC7337812

[7] Gold JAW, Rossen LM, Ahmad FB, . Race, ethnicity, and age trends in persons who died from COVID-19—United States, May-August 2020. MMWR Morb Mortal Wkly Rep. 2020;69(42):1517-1521. doi:10.15585/mmwr.mm6942e1 33090984 PMC7583501

[8] Ahmad FB, Cisewski JA, Miniño A, Anderson RN. Provisional mortality data—United States, 2020. MMWR Morb Mortal Wkly Rep. 2021;70(14):519-522. doi:10.15585/mmwr.mm7014e1 33830988 PMC8030985

[9] Stokes AC, Lundberg DJ, Elo IT, Hempstead K, Bor J, Preston SH. COVID-19 and excess mortality in the United States: a county-level analysis. PLoS Med. 2021;18(5):e1003571. doi:10.1371/journal.pmed.1003571 34014945 PMC8136644

[10] Andrasfay T, Goldman N. Reductions in 2020 US life expectancy due to COVID-19 and the disproportionate impact on the Black and Latino populations. Proc Natl Acad Sci U S A. 2021;118(5):e2014746118. doi:10.1073/pnas.2014746118 33446511 PMC7865122

[11] Goldman N, Andrasfay T. Life expectancy loss among Native Americans during the COVID-19 pandemic. Demogr Res. 2022;47:233-246. doi:10.4054/DemRes.2022.47.9 36506651 PMC9733701

[12] Elo IT, Luck A, Stokes AC, Hempstead K, Xie W, Preston SH. Evaluation of age patterns of COVID-19 mortality by race and ethnicity from March 2020 to October 2021 in the US. JAMA Netw Open. 2022;5(5):e2212686. doi:10.1001/jamanetworkopen.2022.12686 35579900 PMC9115616

[13] Aschmann HE, Riley AR, Chen R, . Dynamics of racial disparities in all-cause mortality during the COVID-19 pandemic. Proc Natl Acad Sci U S A. 2022;119(40):e2210941119. doi:10.1073/pnas.2210941119 36126098 PMC9546535

[14] Truman BI, Chang MH, Moonesinghe R. Provisional COVID-19 age-adjusted death rates, by race and ethnicity —United States, 2020-2021. MMWR Morb Mortal Wkly Rep. 2022;71(17):601-605. doi:10.15585/mmwr.mm7117e2 35482556 PMC9098236

[15] Williams DR, Collins C. Racial residential segregation: a fundamental cause of racial disparities in health. Public Health Rep. 2001;116(5):404-416. doi:10.1016/S0033-3549(04)50068-7 12042604 PMC1497358

[16] Chung-Bridges K, Muntaner C, Fleming LE, . Occupational segregation as a determinant of US worker health. Am J Ind Med. 2008;51(8):555-567. doi:10.1002/ajim.20599 18553362

[17] Siegel M, Sherman R, Li C, Knopov A. The relationship between racial residential segregation and Black-White disparities in fatal police shootings at the city level, 2013-2017. J Natl Med Assoc. 2019;111(6):580-587. doi:10.1016/j.jnma.2019.06.003 31256868

[18] Feagin J, Bennefield Z. Systemic racism and U.S. health care. Soc Sci Med. 2014;103:7-14. doi:10.1016/j.socscimed.2013.09.006 24507906

[19] Widdowson AO, Fisher BW. Mass incarceration and subsequent preventive health care: mechanisms and racial/ethnic disparities. Am J Public Health. 2020;110(S1):S145-S151. doi:10.2105/AJPH.2019.305448 31967899 PMC6987925

[20] Biener AI, Zuvekas SH. Do racial and ethnic disparities in health care use vary with health? Health Serv Res. 2019;54(1):64-74. doi:10.1111/1475-6773.13087 30430571 PMC6338306

[21] Abraham P, Williams E, Bishay AE, Farah I, Tamayo-Murillo D, Newton IG. The roots of structural racism in the United States and their Manifestations during the COVID-19 pandemic. Acad Radiol. 2021;28(7):893-902. doi:10.1016/j.acra.2021.03.025 33994077

[22] Bailey ZD, Krieger N, Agénor M, Graves J, Linos N, Bassett MT. Structural racism and health inequities in the USA: evidence and interventions. Lancet. 2017;389(10077):1453-1463. doi:10.1016/S0140-6736(17)30569-X 28402827

[23] Lundberg I. The gap-closing estimand: a causal approach to study interventions that close disparities across social categories. Sociol Methods Res. Published online January 13, 2022. doi:10.1177/00491241211055769 PMC11823715 39950096

[24] Siegel M, Critchfield-Jain I, Boykin M, Owens A. Actual racial/ethnic disparities in COVID-19 mortality for the non-Hispanic Black compared to non-Hispanic White population in 35 US states and their association with structural racism. J Racial Ethn Health Disparities. 2022;9(3):886-898. doi:10.1007/s40615-021-01028-1 33905110 PMC8077854

[25] Siegel M, Critchfield-Jain I, Boykin M, Owens A, Nunn T, Muratore R. Actual racial/ethnic disparities in COVID-19 mortality for the non-Hispanic Black compared to non-Hispanic White population in 353 US counties and their association with structural racism. J Racial Ethn Health Disparities. 2022;9(5):1697-1725. doi:10.1007/s40615-021-01109-1 34462902 PMC8404537

[26] Tan SB, deSouza P, Raifman M. Structural racism and COVID-19 in the USA: a county-level empirical analysis. J Racial Ethn Health Disparities. 2022;9(1):236-246. doi:10.1007/s40615-020-00948-8 33469868 PMC7815192

[27] Garcia MA, Homan PA, García C, Brown TH. The color of COVID-19: structural racism and the disproportionate impact of the pandemic on older Black and Latinx adults. J Gerontol B Psychol Sci Soc Sci. 2021;76(3):e75-e80. doi:10.1093/geronb/gbaa114 32756973 PMC7454923

[28] Egede LE, Walker RJ. Structural racism, social risk factors, and COVID-19—a dangerous convergence for Black Americans. N Engl J Med. 2020;383(12):e77. doi:10.1056/NEJMp2023616 32706952 PMC7747672

[29] Andraska EA, Alabi O, Dorsey C, . Health care disparities during the COVID-19 pandemic. Semin Vasc Surg. 2021;34(3):82-88. doi:10.1053/j.semvascsurg.2021.08.002 34642040 PMC8349792

[30] Selden TM, Berdahl TA. COVID-19 And racial/ethnic disparities in health risk, employment, and household composition. Health Aff (Millwood). 2020;39(9):1624-1632. doi:10.1377/hlthaff.2020.00897 32663045

[31] Reitsma MB, Claypool AL, Vargo J, . Racial/ethnic disparities in COVID-19 exposure risk, testing, and cases at the subcounty level in California. Health Aff (Millwood). 2021;40(6):870-878. doi:10.1377/hlthaff.2021.00098 33979192 PMC8458028

[32] Forde AT, Crookes DM, Suglia SF, Demmer RT. The weathering hypothesis as an explanation for racial disparities in health: a systematic review. Ann Epidemiol. 2019;33:1-18.e3. doi:10.1016/j.annepidem.2019.02.011 30987864 PMC10676285

[33] Ando W, Horii T, Uematsu T, Hanaki H, Atsuda K, Otori K. Impact of overlapping risks of type 2 diabetes and obesity on coronavirus disease severity in the United States. Sci Rep. 2021;11(1):17968. doi:10.1038/s41598-021-96720-x 34504112 PMC8429758

[34] Rodríguez JE, Campbell KM. Racial and ethnic disparities in prevalence and care of patients with type 2 diabetes. Clin Diabetes. 2017;35(1):66-70. doi:10.2337/cd15-0048 28144049 PMC5241767

[35] Asch DA, Islam MN, Sheils NE, . Patient and hospital factors associated with differences in mortality rates among Black and White US Medicare beneficiaries hospitalized with COVID-19 infection. JAMA Netw Open. 2021;4(6):e2112842. doi:10.1001/jamanetworkopen.2021.12842 34137829 PMC11849740

[36] Goldstein JR, Atherwood S. Improved measurement of racial/ethnic disparities in COVID-19 mortality in the United States. medRxiv. Preprint posted online June 23, 2020. doi:10.1101/2020.05.21.20109116

[37] Paglino E, Lundberg DJ, Cho A, . Monthly excess mortality across counties in the United States during the COVID-19 pandemic, March 2020 to February 2022. medRxiv. Preprint posted online November 21, 2022. doi:10.1101/2022.04.23.22274192 PMC10289647 37352359

[38] Ackley CA, Lundberg DJ, Ma L, Elo IT, Preston SH, Stokes AC. County-level estimates of excess mortality associated with COVID-19 in the United States. SSM Popul Health. 2022;17:101021. doi:10.1016/j.ssmph.2021.101021 35018297 PMC8730693

[39] Parker K, Horowitz JM, Brown A, . What unites and divides urban, suburban and rural communities. Pew Research Center. Accessed March 2, 2023. https://www.pewresearch.org/social-trends/2018/05/22/what-unites-and-divides-urban-suburban-and-rural-communities/

[40] Padamsee TJ, Bond RM, Dixon GN, . Changes in COVID-19 vaccine hesitancy among Black and White individuals in the US. JAMA Netw Open. 2022;5(1):e2144470. doi:10.1001/jamanetworkopen.2021.44470 35061038 PMC8783270

[41] USA Today; Ipsos. Public poll findings and methodology: on COVID-19 vaccine, mask requirements, Americans prioritize common good over personal liberty. Ipsos. Accessed April 10, 2023. https://www.ipsos.com/sites/default/files/ct/news/documents/2021-08/Topline%20USA%20Today%20COVID%20common%20good%20poll%20081821.pdf

[42] Murthy BP, Sterrett N, Weller D, . Disparities in COVID-19 vaccination coverage between urban and rural counties—United States, December 14, 2020-April 10, 2021. MMWR Morb Mortal Wkly Rep. 2021;70(20):759-764. doi:10.15585/mmwr.mm7020e3 34014911 PMC8136424

[43] Wrigley-Field E, Berry KM, Persad G. Race-specific, state-specific COVID-19 vaccination rates adjusted for age. Socius. Published online March 5, 2022. doi:10.1177/23780231221082401 35615692 PMC9128071

[44] National Center for Health Statistics. About provisional mortality statistics, 2018 through last month. Centers for Disease Control and Prevention. Accessed February 6, 2022. https://wonder.cdc.gov/mcd-icd10-provisional.html

[45] Arias E, Schauman WS, Eschbach K, Sorlie PD, Backlund E. The validity of race and Hispanic origin reporting on death certificates in the United States. Vital Health Stat 2. 2008;(148):1-23. 19024798

[46] Arias E, Heron M, Hakes J; National Center for Health Statistics; US Census Bureau. The validity of race and Hispanic-origin reporting on death certificates in the United States: an update. Vital Health Stat 2. 2016;(172):1-21. 28436642

[47] Elo IT, Hendi AS, Ho JY, Vierboom YC, Preston SH. Trends in non-Hispanic White mortality in the United States by metropolitan-nonmetropolitan status and region, 1990-2016. Popul Dev Rev. 2019;45(3):549-583. doi:10.1111/padr.12249 31588154 PMC6771562

[48] US Census Bureau. Methodology for the United States population estimates: vintage 2021, nations, states, counties, and Puerto Rico—April 1, 2020 to July 1, 2021. Accessed March 23, 2023. https://www2.census.gov/programs-surveys/popest/technical-documentation/methodology/2020-2021/methods-statement-v2021.pdf

[49] Bassett MT, Chen JT, Krieger N. Variation in racial/ethnic disparities in COVID-19 mortality by age in the United States: a cross-sectional study. PLoS Med. 2020;17(10):e1003402. doi:10.1371/journal.pmed.1003402 33079941 PMC7575091

[50] Lundberg DJ, Wrigley-Field E, Cho A, . The-uncounted-lab/covid-race-metro. GitHub. Updated April 11, 2023. Accessed April 10, 2023. https://github.com/The-Uncounted-Lab/covid-race-metro/

[51] Leonhardt D. Covid and Race. *The New York Times*. Accessed August 29, 2022. https://www.nytimes.com/2022/06/09/briefing/covid-race-deaths-america.html

[52] Monnat SM. Rural-urban variation in COVID-19 experiences and impacts among U.S. working-age adults. Ann Am Acad Pol Soc Sci. 2021;698(1):111-136. doi:10.1177/00027162211069717 35493266 PMC9055492

[53] James CV, Moonesinghe R, Wilson-Frederick SM, Hall JE, Penman-Aguilar A, Bouye K. Racial/ethnic health disparities among rural adults—United States, 2012-2015. MMWR Surveill Summ. 2017;66(23):1-9. doi:10.15585/mmwr.ss6623a1 29145359 PMC5829953

[54] Warne D, Frizzell LB. American Indian health policy: historical trends and contemporary issues. Am J Public Health. 2014;104 Suppl 3(Suppl 3):S263-S267. doi:10.2105/AJPH.2013.301682 24754649 PMC4035886

[55] Leider JP, Meit M, McCullough JM, . The state of rural public health: enduring needs in a new decade. Am J Public Health. 2020;110(9):1283-1290. doi:10.2105/AJPH.2020.305728 32673103 PMC7427223

[56] Boslett A, Hill E. Mortality during resource booms and busts. J Environ Econ Manage. 2022;115:102696. doi:10.1016/j.jeem.2022.102696 36643912 PMC9835077

[57] Venkataramani AS, Bair EF, O’Brien RL, Tsai AC. Association between automotive assembly plant closures and opioid overdose mortality in the United States: a difference-in-differences analysis. JAMA Intern Med. 2020;180(2):254-262. doi:10.1001/jamainternmed.2019.5686 31886844 PMC6990761

[58] Mueller JT, McConnell K, Burow PB, Pofahl K, Merdjanoff AA, Farrell J. Impacts of the COVID-19 pandemic on rural America. Proc Natl Acad Sci U S A. 2021;118(1):2019378118. doi:10.1073/pnas.2019378118 33328335 PMC7817144

[59] Sanders A, McGranahan D. COVID-19 vaccinations in rural America. US Department of Agriculture Economic Research Service. Accessed July 14, 2022. https://www.ers.usda.gov/covid-19/rural-america/covid-19-vaccinations-in-rural-america/

[60] Barro RJ. Vaccination rates and COVID outcomes across U.S. States. Accessed March 23, 2023. https://www.nber.org/system/files/working_papers/w29884/w29884.pdf 10.1016/j.ehb.2022.101201 PMC9933209 36434953

[61] Watson OJ, Barnsley G, Toor J, Hogan AB, Winskill P, Ghani AC. Global impact of the first year of COVID-19 vaccination: a mathematical modelling study. Lancet Infect Dis. 2022;22(9):1293-1302. doi:10.1016/S1473-3099(22)00320-6 35753318 PMC9225255

[62] Persad G, Peek ME, Emanuel EJ. Fairly prioritizing groups for access to COVID-19 vaccines. JAMA. 2020;324(16):1601-1602. doi:10.1001/jama.2020.18513 32910182

[63] Johnson JH Jr, Bonds JM, Parnell AM, Bright CM. Coronavirus vaccine distribution: moving to a race conscious approach for a racially disparate problem. J Racial Ethn Health Disparities. 2021;8(4):799-802. doi:10.1007/s40615-021-01051-2 33948908 PMC8095217

[64] Kriss JL, Hung MC, Srivastav A, . COVID-19 vaccination coverage, by race and ethnicity—National Immunization Survey Adult COVID Module, United States, December 2020-November 2021. MMWR Morb Mortal Wkly Rep. 2022;71(23):757-763. doi:10.15585/mmwr.mm7123a2 35679179 PMC9181054

[65] Wrigley-Field E, Berry KM, Stokes AC, Leider JP. “Pandemic of the unvaccinated”: at midlife, White people are less vaccinated but still at less risk of COVID-19 mortality in Minnesota. medRxiv. Preprint posted online June 17, 2022. doi:10.1101/2022.03.02.22271808

[66] Bajaj SS, Stanford FC. Beyond Tuskegee—vaccine distrust and everyday racism. N Engl J Med. 2021;384(5):e12. doi:10.1056/NEJMpv2035827 33471971 PMC9908408

[67] Nguyen LH, Joshi AD, Drew DA, ; COPE Consortium. Self-reported COVID-19 vaccine hesitancy and uptake among participants from different racial and ethnic groups in the United States and United Kingdom. Nat Commun. 2022;13(1):636. doi:10.1038/s41467-022-28200-3 35105869 PMC8807721

[68] Centers for Disease Control and Prevention. COVID Data Tracker. Accessed February 25, 2023. https://covid.cdc.gov/covid-data-tracker/#vaccination-demographics-trends

[69] Dada D, Djiometio JN, McFadden SM, . Strategies that promote equity in COVID-19 vaccine uptake for Black communities: a review. J Urban Health. 2022;99(1):15-27. doi:10.1007/s11524-021-00594-3 35018612 PMC8751469

[70] Privor-Dumm L, King T. Community-based strategies to engage pastors can help address vaccine hesitancy and health disparities in Black communities. J Health Commun. 2020;25(10):827-830. doi:10.1080/10810730.2021.1873463 33719889

[71] Wong CA, Dowler S, Moore AF, . COVID-19 vaccine administration, by race and ethnicity—North Carolina, December 14, 2020-April 6, 2021. MMWR Morb Mortal Wkly Rep. 2021;70(28):991-996. doi:10.15585/mmwr.mm7028a2 34264909 PMC8314707

[72] Quinn SC, Andrasik MP. Addressing vaccine hesitancy in BIPOC communities—toward trustworthiness, partnership, and reciprocity. N Engl J Med. 2021;385(2):97-100. doi:10.1056/NEJMp2103104 33789007

[73] Prusaczyk B. Strategies for disseminating and implementing COVID-19 vaccines in rural areas. Open Forum Infect Dis. 2021;8(6):ofab152. doi:10.1093/ofid/ofab152 34183979 PMC8083584

[74] AlSayyad Y, DeCruz R, Levine JS. An Alabama woman’s neighborly vaccination campaign. Accessed August 29, 2022. *The New Yorker*. https://www.newyorker.com/culture/the-new-yorker-documentary/an-alabama-womans-neighborly-vaccination-campaign

[75] Wrigley-Field E, Kiang MV, Riley AR, . Geographically targeted COVID-19 vaccination is more equitable and averts more deaths than age-based thresholds alone. Sci Adv. 2021;7(40):eabj2099. doi:10.1126/sciadv.abj2099 34586843 PMC8480919

[76] Chen YH, Glymour M, Riley A, . Excess mortality associated with the COVID-19 pandemic among Californians 18-65 years of age, by occupational sector and occupation: March through November 2020. PLoS One. 2021;16(6):e0252454. doi:10.1371/journal.pone.0252454 34086762 PMC8177528

[77] Riley AR, Chen YH, Matthay EC, . Excess mortality among Latino people in California during the COVID-19 pandemic. SSM Popul Health. 2021;15:100860. doi:10.1016/j.ssmph.2021.100860 34307826 PMC8283318

[78] Sandoval-Olascoaga S, Venkataramani AS, Arcaya MC. Eviction moratoria expiration and COVID-19 infection risk across strata of health and socioeconomic status in the United States. JAMA Netw Open. 2021;4(8):e2129041. doi:10.1001/jamanetworkopen.2021.29041 34459904 PMC8406080

[79] Matthay EC, Duchowny KA, Riley AR, Galea S. Projected all-cause deaths attributable to COVID-19-related unemployment in the United States. Am J Public Health. 2021;111(4):696-699. doi:10.2105/AJPH.2020.306095 33600244 PMC7958047

[80] Bassett MT, Galea S. Reparations as a public health priority—a strategy for ending Black-White health disparities. N Engl J Med. 2020;383(22):2101-2103. doi:10.1056/NEJMp2026170 33031653

[81] Gilbert KL, Ray R. Why police kill Black males with impunity: applying public health critical race praxis (PHCRP) to address the determinants of policing behaviors and “justifiable” homicides in the USA. J Urban Health. 2016;93(Suppl 1)(suppl 1):122-140. doi:10.1007/s11524-015-0005-x 26661386 PMC4824696

[82] Bor J, Stokes AC, Raifman J, . Missing Americans: early death in the United States, 1933-2021. medRxiv. Preprint posted online July 12, 2022. doi:10.1101/2022.06.29.22277065 PMC10257439 37303714

[83] Hamed S, Bradby H, Ahlberg BM, Thapar-Björkert S. Racism in healthcare: a scoping review. BMC Public Health. 2022;22(1):988. doi:10.1186/s12889-022-13122-y 35578322 PMC9112453

[84] Powe NR, Cooper LA. Diversifying the racial and ethnic composition of the physician workforce. Ann Intern Med. 2004;141(3):223-224. doi:10.7326/0003-4819-141-3-200408030-00013 15289221

[85] Zalla LC, Martin CL, Edwards JK, Gartner DR, Noppert GA. A geography of risk: structural racism and coronavirus disease 2019 mortality in the United States. Am J Epidemiol. 2021;190(8):1439-1446. doi:10.1093/aje/kwab059 33710272 PMC7989642

[86] Spencer MR, Ahmad F. National vital statistics rapid release: timeliness of death certificate data for mortality surveillance and provisional estimates. Centers for Disease Control and Prevention. Accessed March 23, 2023. https://www.cdc.gov/nchs/data/vsrr/report001.pdf

[87] Goldstein JR, Morning AJ. The multiple-race population of the United States: issues and estimates. Proc Natl Acad Sci U S A. 2000;97(11):6230-6235. doi:10.1073/pnas.100086897 10811886 PMC18587

[88] Gullickson A, Morning A. Choosing race: multiracial ancestry and identification. Soc Sci Res. 2011;40(2):498-512. doi:10.1016/j.ssresearch.2010.12.010

[89] Paglino E, Lundberg DJ, Zhou Z, . Differences between reported COVID-19 deaths and estimated excess deaths in counties across the United States, March 2020 to February 2022. medRxiv. Preprint posted online January 18, 2023. doi:10.1101/2023.01.16.23284633

