# Supplementary material for: COVID-19 Mortality by Race and Ethnicity in US Metropolitan and Nonmetropolitan Areas, March 2020 to February 2022
Source: JAMA Netw Open. 2023 May 2;6(5):e2311098. doi: 10.1001/jamanetworkopen.2023.11098 (PMC10155069; doi:10.1001/jamanetworkopen.2023.11098)
Supplement: Supplement 1. — eTable 1. Proportion of Population and Adult COVID-19 Deaths by Metropolitan and Nonmetropolitan Area eTable 2. Changes in Adult COVID-19 Death Rates From Initial to Second Wave eTable 3. Changes in Adult COVID-19 Death Rates From Initial to Alpha Wave eTable 4. Changes in Adult COVID-19 Death Rates From First to Second Year of Pandemic eTable 5. Full Results for the Demographic Decomposition of the Components Contributing to National Changes in Disparities in COVID-19 Mortality for Hispanic and Non-Hispanic Black Adults vs Non-Hispanic White Adults From Initial to Subsequent Waves eAppendix 1. Data-Querying Approach eAppendix 2. Age-Standardization Procedure eAppendix 3. Decomposition Analysis Derivation and Formulas eReferences. [file jamanetwopen-e2311098-s001.pdf]

Supplemental Online Content

Lundberg DJ, Wrigley-Field E, Cho A, et al. COVID-19 mortality by race and ethnicity in US metropolitan and nonmetropolitan areas, March 2020 to February 2022. *JAMA Netw Open*. 2023;6(5):e2311098. doi:10.1001/jamanetworkopen.2023.11098

eTable 1. Proportion of Population and Adult COVID-19 Deaths by Metropolitan and Nonmetropolitan Area

eTable 2. Changes in Adult COVID-19 Death Rates From Initial to Second Wave

eTable 3. Changes in Adult COVID-19 Death Rates From Initial to Alpha Wave

eTable 4. Changes in Adult COVID-19 Death Rates From First to Second Year of Pandemic

eTable 5. Full Results for the Demographic Decomposition of the Components Contributing to National Changes in Disparities in COVID-19 Mortality for Hispanic and Non-Hispanic Black Adults vs Non-Hispanic White Adults From Initial to Subsequent Waves

eAppendix 1. Data-Querying Approach

eAppendix 2. Age-Standardization Procedure

eAppendix 3. Decomposition Analysis Derivation and Formulas

eReferences.

This supplemental material has been provided by the authors to give readers additional information about their work.

© 2023 Lundberg DJ et al. *JAMA Network Open.*

eTable 1. Proportion of Population and Adult COVID-19 Deaths by Metropolitan and Nonmetropolitan Area

Racial and Ethnic Groups	Metro-Nonmetro Categories	Proportion of the Population Residing in an Area During:		Proportion of COVID-19 Deaths that Were in an Area During:				
		2020	2021	Initial Wave	Second Wave	Alpha Wave	Delta Wave	Omicron Wave
Hispanic	Large Metros	68.6%	68.3%	88.7%	61.6%	66.7%	59.6%	60.6%
	Medium/Small Metros	25.2%	25.4%	9.5%	31.3%	25.9%	29.8%	30.1%
	Nonmetro Areas	6.3%	6.3%	1.8%	7.1%	7.4%	10.6%	9.3%
Non-Hispanic Black	Large Metros	67.0%	66.9%	82.2%	53.7%	59.2%	55.5%	65.0%
	Medium/Small Metros	24.2%	24.3%	12.5%	28.9%	26.8%	30.4%	24.7%
	Nonmetro Areas	8.8%	8.8%	5.3%	17.4%	14.1%	14.1%	10.3%
Non-Hispanic White	Large Metros	49.3%	49.0%	71.8%	46.7%	40.6%	33.9%	40.4%
	Medium/Small Metros	32.9%	33.1%	21.7%	34.9%	34.2%	37.9%	34.1%
	Nonmetro Areas	17.8%	17.9%	6.5%	18.5%	25.2%	28.2%	25.6%
All Racial and Ethnic Groups	Large Metros	56.4%	56.2%	78.0%	52.8%	48.7%	41.1%	46.4%
	Medium/Small Metros	29.6%	29.8%	16.6%	32.1%	31.1%	35.5%	32.1%
	Nonmetro Areas	14.0%	14.0%	5.4%	15.1%	20.2%	23.4%	21.5%

Notes: The first two years of the pandemic (March 1, 2020 to February 28, 2022) were divided into 5 pandemic waves: the initial wave (March 1 through May 31, 2020), the second wave (June 1 through August 31, 2020), the Alpha wave (September 1, 2020 through May 31, 2021), the Delta wave (June 1 through October 31, 2021), and the Omicron wave (November 1, 2021 through February 28, 2022). The 2020 and 2021 mid-year Census population estimates were used to calculate the population proportions. Adult COVID-19 deaths referred to deaths among adults 25 years and older.

© 2023 Lundberg DJ et al. *JAMA Network Open.*

eTable 2. Changes in Adult COVID-19 Death Rates From Initial to Second Wave

Metro-Nonmetro Category	Race and Ethnicity	ASDR		% Change in ASDR between Initial and Second Waves (95% CI)	RR (95% CI)		% Change in RR between Initial and Second Waves (95% CI)
		Initial Wave	Second Wave		Initial Wave	Second Wave	
Large Metro	Hispanic	403.0	326.9	-18.9 (-21.0 to -16.8)	1.9 (1.9 to 2.0)	3.7 (3.6 to 3.7)	90.0 (84.4 to 95.6)
	Non-Hispanic AI/AN	122.3	219.0	79.1 (48.5 to 109.7)	0.6 (0.5 to 0.7)	2.5 (2.1 to 2.8)	319.6 (222.7 to 416.6)
	Non-Hispanic Asian	240.0	87.3	-63.6 (-66.8 to -60.4)	1.1 (1.1 to 1.2)	1.0 (0.9 to 1.0)	-14.7 (-19.5 to -10.0)
	Non-Hispanic Black	590.7	205.7	-65.2 (-66.7 to -63.6)	2.8 (2.8 to 2.9)	2.3 (2.2 to 2.4)	-18.4 (-21.0 to -15.8)
	Non-Hispanic White	209.3	89.3	-57.3 (-58.5 to -56.2)	Ref	Ref	Ref
Medium/Small Metro	Hispanic	120.3	461.4	283.4 (272.9 to 294.0)	1.4 (1.3 to 1.5)	5.0 (4.8 to 5.1)	260.6 (239.1 to 282.1)
	Non-Hispanic AI/AN	233.1	234.6	0.7 (-16.5 to 17.9)	2.7 (2.4 to 3.0)	2.5 (2.2 to 2.8)	-5.3 (-21.7 to 11.1)
	Non-Hispanic Asian	63.9	89.8	40.5 (23.2 to 57.8)	0.7 (0.7 to 0.8)	1.0 (0.9 to 1.1)	32.1 (12.5 to 51.7)
	Non-Hispanic Black	249.2	310.9	24.7 (19.6 to 29.9)	2.9 (2.8 to 3.0)	3.4 (3.2 to 3.5)	17.3 (11.2 to 23.5)
	Non-Hispanic White	86.9	92.4	6.3 (3.8 to 8.9)	Ref	Ref	Ref
Nonmetro	Hispanic	83.1	405.8	388.5 (361.2 to 415.9)	1.8 (1.6 to 2.0)	4.7 (4.4 to 4.9)	161.1 (127.3 to 194.8)
	Non-Hispanic AI/AN	273.8	463.6	69.3 (52.8 to 85.8)	5.9 (5.3 to 6.5)	5.3 (4.9 to 5.8)	-9.5 (-21.6 to 2.5)
	Non-Hispanic Asian	31.8	62.0	95.1 (24.9 to 165.4)	0.7 (0.4 to 1.0)	0.7 (0.5 to 0.9)	4.3 (-48.4 to 56.9)
	Non-Hispanic Black	256.4	451.7	76.2 (67.4 to 84.9)	5.5 (5.2 to 5.9)	5.2 (4.9 to 5.4)	-5.9 (-13.1 to 1.4)
	Non-Hispanic White	46.5	87.1	87.1 (81.7 to 92.6)	Ref	Ref	Ref
All Areas	Hispanic	311.9	365.7	17.2 (15.0 to 19.4)	2.2 (2.2 to 2.3)	4.1 (4.0 to 4.1)	82.5 (78.1 to 86.9)
	Non-Hispanic AI/AN	217.7	318.8	46.4 (35.2 to 57.6)	1.6 (1.4 to 1.7)	3.5 (3.3 to 3.8)	128.0 (106.6 to 149.3)
	Non-Hispanic Asian	205.7	87.2	-57.6 (-60.8 to -54.5)	1.5 (1.4 to 1.5)	1.0 (0.9 to 1.0)	-34.0 (-37.3 to -30.7)
	Non-Hispanic Black	478.5	252.9	-47.2 (-48.7 to -45.6)	3.4 (3.4 to 3.5)	2.8 (2.8 to 2.9)	-17.7 (-19.7 to -15.7)
	Non-Hispanic White	140.0	89.9	-35.8 (-36.8 to -34.7)	Ref	Ref	Ref

AI/AN = American Indian and Alaska Native | ASDR = Age-Standardized Death Rates, Deaths per 100,000 Person-Years | RR = Rate Ratio | Ref = Reference group

Notes: We examined differences in age-standardized COVID-19 death rates among adults aged 25 and older by race and ethnicity across metropolitan and nonmetropolitan areas. We compared the initial wave of the pandemic (March 1 through May 31, 2020) to the second wave (June 1 through August 31, 2020). Death rates were standardized using 3 age groups (25-54, 55-74 and 75+) and the overall U.S. 2020 population as the standard population. To facilitate comparison across waves of different durations, we annualized the death rates and report the estimates in units of COVID-19 deaths per 100,000 adult person-years.

© 2023 Lundberg DJ et al. *JAMA Network Open.*

eTable 3. Changes in Adult COVID-19 Death Rates From Initial to Alpha Wave

Metro-Nonmetro Category	Race and Ethnicity	ASDR		% Change in ASDR between Initial and Alpha Waves (95% CI)	RR (95% CI)		% Change in RR between Initial and Alpha Waves (95% CI)
		Initial Wave	Alpha Wave		Initial Wave	Alpha Wave	
Large Metro	Hispanic	403.0	362.0	-10.2 (-12.0 to -8.4)	1.9 (1.9 to 2.0)	2.0 (2.0 to 2.0)	4.3 (2.1 to 6.6)
	Non-Hispanic AI/AN	122.3	260.5	113.0 (88.9 to 137.1)	0.6 (0.5 to 0.7)	1.4 (1.3 to 1.5)	147.4 (98.0 to 196.9)
	Non-Hispanic Asian	240.0	182.8	-23.8 (-26.9 to -20.8)	1.1 (1.1 to 1.2)	1.0 (1.0 to 1.0)	-11.5 (-14.6 to -8.5)
	Non-Hispanic Black	590.7	258.6	-56.2 (-57.7 to -54.8)	2.8 (2.8 to 2.9)	1.4 (1.4 to 1.5)	-49.2 (-50.2 to -48.1)
	Non-Hispanic White	209.3	180.2	-13.9 (-15.0 to -12.8)	Ref	Ref	Ref
Medium/Small Metro	Hispanic	120.3	388.9	223.2 (216.3 to 230.1)	1.4 (1.3 to 1.5)	1.9 (1.8 to 1.9)	34.1 (26.8 to 41.4)
	Non-Hispanic AI/AN	233.1	408.5	75.3 (60.0 to 90.6)	2.7 (2.4 to 3.0)	2.0 (1.8 to 2.1)	-27.3 (-37.1 to -17.5)
	Non-Hispanic Asian	63.9	150.6	135.5 (120.7 to 150.4)	0.7 (0.7 to 0.8)	0.7 (0.7 to 0.7)	-2.3 (-14.1 to 9.5)
	Non-Hispanic Black	249.2	327.6	31.5 (27.3 to 35.6)	2.9 (2.8 to 3.0)	1.6 (1.5 to 1.6)	-45.5 (-47.8 to -43.1)
	Non-Hispanic White	86.9	209.4	141.0 (138.7 to 143.4)	Ref	Ref	Ref
Nonmetro	Hispanic	83.1	430.9	418.8 (400.3 to 437.3)	1.8 (1.6 to 2.0)	1.6 (1.5 to 1.6)	-12.2 (-22.7 to -1.7)
	Non-Hispanic AI/AN	273.8	620.0	126.4 (113.1 to 139.8)	5.9 (5.3 to 6.5)	2.3 (2.2 to 2.3)	-61.7 (-66.0 to -57.4)
	Non-Hispanic Asian	31.8	144.9	355.9 (291.6 to 420.3)	0.7 (0.4 to 1.0)	0.5 (0.5 to 0.6)	-22.8 (-55.6 to 9.9)
	Non-Hispanic Black	256.4	422.6	64.8 (58.3 to 71.4)	5.5 (5.2 to 5.9)	1.5 (1.5 to 1.6)	-72.1 (-74.0 to -70.3)
	Non-Hispanic White	46.5	274.9	490.9 (485.4 to 496.5)	Ref	Ref	Ref
All Areas	Hispanic	311.9	373.2	19.6 (17.9 to 21.4)	2.2 (2.2 to 2.3)	1.8 (1.8 to 1.8)	-19.0 (-20.5 to -17.4)
	Non-Hispanic AI/AN	217.7	448.7	106.1 (96.8 to 115.4)	1.6 (1.4 to 1.7)	2.2 (2.1 to 2.2)	39.6 (28.7 to 50.4)
	Non-Hispanic Asian	205.7	176.5	-14.2 (-17.2 to -11.2)	1.5 (1.4 to 1.5)	0.9 (0.8 to 0.9)	-41.9 (-43.8 to -40.0)
	Non-Hispanic Black	478.5	289.8	-39.4 (-40.8 to -38.1)	3.4 (3.4 to 3.5)	1.4 (1.4 to 1.4)	-59.0 (-59.7 to -58.3)
	Non-Hispanic White	140.0	206.7	47.7 (46.7 to 48.7)	Ref	Ref	Ref

AI/AN = American Indian and Alaska Native | ASDR = Age-Standardized Death Rates, Deaths per 100,000 Person-Years | RR = Rate Ratio | Ref = Reference group

Notes: We examined differences in age-standardized COVID-19 death rates among adults aged 25 years and older by race and ethnicity across metropolitan and nonmetropolitan areas. We compared the initial wave of the pandemic (March 1 through May 31, 2020) to the Alpha wave (September 1, 2020 through May 31, 2021). Death rates were standardized using 3 age groups (25-54, 55-74 and 75+) and the overall U.S. 2020 population as the standard population. To facilitate comparison across waves of different durations, we annualized the death rates and report the estimates in units of COVID-19 deaths per 100,000 adult person-years.

© 2023 Lundberg DJ et al. *JAMA Network Open.*

eTable 4. Changes in Adult COVID-19 Death Rates From First to Second Year of Pandemic

Metro-Nonmetro Category	Race and Ethnicity	ASDR First Year	ASDR Second Year	% Change in ASDR between the First and Second Years (95% CI)	RR (95% CI) First Year	RR (95% CI) Second Year	% Change in RR between the First and Second Years (95% CI)
Large Metro	Hispanic	416.4	199.7	-52.1 (-53.0 to -51.1)	2.2 (2.2 to 2.2)	1.3 (1.3 to 1.4)	-39.2 (-40.1 to -38.2)
	Non-Hispanic AI/AN	255.5	218.6	-14.4 (-22.9 to -5.9)	1.3 (1.3 to 1.4)	1.5 (1.4 to 1.6)	8.6 (-1.4 to 18.5)
	Non-Hispanic Asian	198.7	81.1	-59.2 (-60.9 to -57.4)	1.0 (1.0 to 1.1)	0.5 (0.5 to 0.6)	-48.2 (-49.7 to -46.8)
	Non-Hispanic Black	347.2	234.3	-32.5 (-33.6 to -31.4)	1.8 (1.8 to 1.8)	1.6 (1.5 to 1.6)	-14.4 (-15.7 to -13.1)
	Non-Hispanic NH/OPI	381.0	371.5	-2.5 (-14.3 to 9.3)	2.0 (1.8 to 2.2)	2.5 (2.3 to 2.7)	23.7 (8.9 to 38.5)
	Non-Hispanic White	189.6	149.4	-21.2 (-21.9 to -20.5)	Ref	Ref	Ref
Medium/Small Metro	Hispanic	404.9	247.8	-38.8 (-40.4 to -37.2)	2.2 (2.2 to 2.2)	1.3 (1.3 to 1.3)	-42.2 (-43.5 to -40.9)
	Non-Hispanic AI/AN	394.3	301.5	-23.5 (-29.6 to -17.5)	2.2 (2.1 to 2.3)	1.6 (1.5 to 1.6)	-27.8 (-32.8 to -22.7)
	Non-Hispanic Asian	139.0	100.9	-27.4 (-32.3 to -22.5)	0.8 (0.7 to 0.8)	0.5 (0.5 to 0.5)	-31.4 (-35.4 to -27.4)
	Non-Hispanic Black	346.0	277.1	-19.9 (-21.8 to -18.0)	1.9 (1.9 to 1.9)	1.4 (1.4 to 1.5)	-24.3 (-26.1 to -22.6)
	Non-Hispanic NH/OPI	254.1	282.7	11.3 (-4.1 to 26.7)	1.4 (1.2 to 1.5)	1.5 (1.3 to 1.6)	5.1 (-10.3 to 20.5)
	Non-Hispanic White	182.6	193.3	5.9 (5.0 to 6.7)	Ref	Ref	Ref
Nonmetro	Hispanic	417.0	306.9	-26.4 (-29.6 to -23.2)	1.9 (1.9 to 2.0)	1.2 (1.2 to 1.2)	-37.4 (-39.9 to -35.0)
	Non-Hispanic AI/AN	617.8	373.6	-39.5 (-43.7 to -35.3)	2.8 (2.7 to 2.9)	1.5 (1.4 to 1.5)	-48.6 (-51.4 to -45.8)
	Non-Hispanic Asian	125.3	108.9	-13.1 (-26.8 to 0.7)	0.6 (0.5 to 0.6)	0.4 (0.4 to 0.5)	-26.1 (-37.0 to -15.2)
	Non-Hispanic Black	455.6	307.5	-32.5 (-35.0 to -30.0)	2.1 (2.0 to 2.1)	1.2 (1.2 to 1.2)	-42.6 (-44.5 to -40.8)
	Non-Hispanic NH/OPI	153.8	232.7	51.3 (10.4 to 92.2)	0.7 (0.5 to 0.9)	0.9 (0.7 to 1.1)	28.6 (-14.6 to 71.8)
	Non-Hispanic White	217.9	256.3	17.6 (16.5 to 18.7)	Ref	Ref	Ref
All Areas	Hispanic	413.5	218.7	-47.1 (-47.9 to -46.3)	2.2 (2.1 to 2.2)	1.2 (1.2 to 1.2)	-44.4 (-45.1 to -43.8)
	Non-Hispanic AI/AN	441.8	306.1	-30.7 (-33.9 to -27.5)	2.3 (2.2 to 2.4)	1.7 (1.6 to 1.7)	-27.2 (-30.0 to -24.4)
	Non-Hispanic Asian	187.0	85.1	-54.5 (-56.1 to -52.9)	1.0 (1.0 to 1.0)	0.5 (0.5 to 0.5)	-52.2 (-53.4 to -51.0)
	Non-Hispanic Black	356.5	251.1	-29.5 (-30.5 to -28.6)	1.9 (1.8 to 1.9)	1.4 (1.4 to 1.4)	-26.0 (-26.9 to -25.1)
	Non-Hispanic NH/OPI	306.5	321.3	4.8 (-4.3 to 13.9)	1.6 (1.5 to 1.7)	1.8 (1.6 to 1.9)	10.1 (0.3 to 20.0)
	Non-Hispanic White	192.3	183.1	-4.8 (-5.3 to -4.3)	Ref	Ref	Ref

AI/AN = American Indian and Alaska Native | NH/OPI = Native Hawaiian and Other Pacific Islander | ASDR = Age-Standardized Death Rates, Deaths per 100,000 Person-Years | RR = Rate Ratio | Ref = Reference group

Notes: We examined differences in age-standardized COVID-19 death rates among adults aged 25 years and older by race and ethnicity across metropolitan and nonmetropolitan areas. We compared the first year of the pandemic (March 1, 2020 through February 28, 2021) to the second year (March 1, 2021 through February 28, 2022). Death rates were standardized using 3 age groups (25-54, 55-74 and 75+) and the overall U.S. 2020 population as the standard population. Estimates for non-Hispanic NH/OPI adults have larger confidence intervals than the other racial and ethnic groups in the table and should be interpreted with caution.

© 2023 Lundberg DJ et al. *JAMA Network Open.*

eTable 5. Full Results for the Demographic Decomposition of the Components Contributing to National Changes in Disparities in COVID-19 Mortality for Hispanic and Non-Hispanic Black Adults vs Non-Hispanic White Adults From Initial to Subsequent Waves

	**Decomposition Components, Deaths per 100,000 Person-Years**				
	**a**	**b**	**c**	**d**	**Total**
**Decomposing the National Decrease in Disparities for Hispanic compared to Non-Hispanic White Adults**					
**Initial vs. Second Wave**	109.6	58.1	-64.0	0.1	103.8
**Initial vs. Alpha Wave**	110.5	-54.9	-61.1	0.0	-5.5
**Initial vs. Delta Wave**	-48.4	-17.3	-72.3	0.1	-137.9
**Initial vs. Omicron Wave**	31.5	-119.6	-72.4	0.2	-160.3
**Decomposing the National Decrease in Disparities for Non-Hispanic Black compared to Non-Hispanic White Adults**					
**Initial vs. Second Wave**	-163.6	58.1	-70.1	0.0	-175.5
**Initial vs. Alpha Wave**	-133.8	-54.9	-66.7	-0.1	-255.5
**Initial vs. Delta Wave**	-179.0	-17.3	-71.3	-0.2	-267.8
**Initial vs. Omicron Wave**	-116.1	-119.6	-57.5	-0.2	-293.4

Notes: We conducted a demographic decomposition analysis using age-standardized COVID-19 death rates among adults 25 years and older by race and ethnicity across metropolitan and nonmetropolitan areas. We sought to understand the contribution of different mechanisms to national reductions in disparities for Hispanic and non-Hispanic Black adults compared to non-Hispanic White adults between the initial wave of the pandemic and the subsequent waves. The waves in the analysis were: the initial wave (March 1 through May 31, 2020), the second wave (June 1 through August 31, 2020), the Alpha wave (September 1, 2020 through May 31, 2021), the Delta wave (June 1 through October 31, 2021), and the Omicron wave (November 1, 2021 through February 28, 2022). To facilitate comparison across waves of different durations, we annualized the death rates and report the estimates in units of COVID-19 deaths per 100,000 adult person-years. The components examined within the decomposition were: (a) the geographically-standardized decrease in Hispanic or non-Hispanic Black mortality rates, (b) the geographically-standardized increase in non-Hispanic White mortality rates, (c) the mortality effect of shifts in where deaths occurred from metropolitan to nonmetropolitan areas, and (d) the mortality effect of shifts in the racial and ethnic population composition in metropolitan and nonmetropolitan areas. The 4 components sum to the total national change in disparities. The national changes in disparities were all decreases except for the change in disparity for Hispanic compared to non-Hispanic White adults between the initial and second waves which was an increase. Formulas for the decomposition and their derivations are available in the **Appendix**.

© 2023 Lundberg DJ et al. *JAMA Network Open.*

eAppendix 1. Data-Querying Approach

Data Source: CDC WONDER (https://wonder.cdc.gov/mcd-icd10-provisional.html)

Rationale for Querying Approach: It is possible to sort CDC WONDER data by variables such as race, ethnicity, 10-year age groups, urbanization, region, month, and year in one query. However, sorting in this way puts the data into smaller cells than needed for this study, resulting in unnecessary data suppression because death counts between 0 and 9 are censored. To limit data suppression, our approach was to submit multiple queries at every level of aggregation needed in the study. In total, we completed 162 queries.

Query #1 (Pandemic Year Data): This set of queries outputs COVID-19 death counts by pandemic year, urbanization, age, race and ethnicity. 36 files are generated.

- Use Multiple Cause Provisional Mortality Statistics, 2018 through Last Month
- Select Multiple Cause of Death (Any Mention on Death Certificate): Covid-19 (U07.1)
- Select Show Zero Values and Suppressed Values
- Select Months (Separate Files for Each)
    - March 2020 through February 2021
    - March 2021 through February 2022
- Select 2013 Urbanization (by Residence) Categories (Separate Files for Each)
    - Nonmetro (Noncore (Nonmetro) and Micropolitan (Nonmetro))
    - Small/Medium Metro (Small Metro and Medium Metro)
    - Large Metro (Large Fringe Metro and Large Central Metro)
- Select 10-Year Age Categories (Separate Files for Each)
    - Ages 25-54
    - Ages 55-74
    - Ages 75+
- Select Race and Ethnicity (Separate files for Each)
    - Select Hispanic and All Races
    - Select Non-Hispanic and All Races and Group by Single Race 6

Query #2 (Pandemic Wave Data): This set of queries outputs COVID-19 death counts by pandemic wave, urbanization, age, race and ethnicity. 90 files are generated.

- Use Multiple Cause Provisional Mortality Statistics, 2018 through Last Month
- Select Multiple Cause of Death (Any Mention on Death Certificate): Covid-19 (U07.1)
- Select Show Zero Values and Suppressed Values
- Select Months (Separate Files for Each)
    - March 2020 through May 2020
    - June through August 2020
    - September 2020 through May 2021
    - June through October 2021
    - November 2021 through February 2022
- Select 2013 Urbanization (By Residence) Categories (Separate Files for Each)
    - Nonmetro (Noncore (Nonmetro) and Micropolitan (Nonmetro))
    - Small/Medium Metro (Small Metro and Medium Metro)
    - Large Metro (Large Fringe Metro and Large Central Metro)
- Select 10-Year Age Categories (Separate Files for Each)
    - Ages 25-54

© 2023 Lundberg DJ et al. *JAMA Network Open.*

  - Ages 55-74
  - Ages 75+
- Select Race and Ethnicity (Separate files for Each)
  - Select Hispanic and All Races
  - Select Non-Hispanic and All Races and Group by Single Race 6

Query #3 (Monthly Data): This set of queries outputs COVID-19 death counts by month, urbanization, age, race and ethnicity. 36 files are generated.

- Use Multiple Cause Provisional Mortality Statistics, 2018 through Last Month
- Select Multiple Cause of Death (Any Mention on Death Certificate): Covid-19 (U07.1)
- Select Show Zero Values and Suppressed Values
- Select Months March 2020 through February 2022
- Select 2013 Urbanization (By Residence) Categories (Separate Files for Each)
  - Nonmetro (Noncore (Nonmetro) and Micropolitan (Nonmetro))
  - Small/Medium Metro (Small Metro and Medium Metro)
  - Large Metro (Large Fringe Metro and Large Central Metro)
- Select 10-Year Age Categories (Separate Files for Each)
  - Ages 25-54
  - Ages 55-74
  - Ages 75+
- Select Race and Ethnicity (Separate files for Each)
  - Select Hispanic and All Races
  - Select Non-Hispanic and Black or African American
  - Select Non-Hispanic and White
  - Select All Races and Ethnicities
- Group by Month

© 2023 Lundberg DJ et al. *JAMA Network Open.*

eAppendix 2. Age-Standardization Procedure

The U.S. Census 2020 population was used as the standard population. For each geographic and temporal unit, age-specific death rates were calculated for ages 25-54, 55-74, and 75+. Each age-specific death rate was multiplied by the corresponding percentage of the standard population in that age group, and then the 3 components were added together to produce the age-standardized death rate. It was not possible to use 10-year age intervals for age-standardization due to data suppression.

We calculated the variance for our estimates of age-standardized death rates, relative changes in rates, rate ratios, and relative changes in rate ratios using the following approach.

Variance for Metro-Specific Age-Standardized Death Rates:
The variances of the age standardized death rates (ASDRs) were calculated using the method described in the CDC Vital Statistics of the United States Technical Appendix with the following formula:[1]

$$var(ASDR) = \sum {w_i}^2 {R_i}^2 (\frac{1}{D_i})$$

Where $ASDR$ is an age standardized death rate, i is the age group (25-54, 55-74, and 75+), w_i is the age specific weight based on the 2020 Census population age distribution, R_i is the age specific death rate and D_i is the number of deaths used to calculate the age specific death rate. These values were used to calculate the confidence intervals of the relative change, rate ratio, and relative change in rate ratio and were not reported, in reference to a report from Truman et. al.[2]

Variance for All Areas Population Weighted Average Age-Standardized Death Rates:
The variance of the all areas population weighted average ASDRs were calculated using the following formula:[1]

$$var(PWA) = \sum {p_j}^2 \times var(ASDRj)$$

Where *PWA* the population weighted average, *j* is the metro category, p_j is the metro specific population weight and $ASDR_j$ is the metro-specific ASDR.

Variance for Relative Change in Age-Standardized Death Rates:
The variances of each year were used to calculate the 95% confidence interval of the relative change in ASDRs and this interval was divided by the 2020 ASDR to get the percent change with the following formula:

$$\frac{(R_{21} - R_{20}) \pm 1.96 * \sqrt{var(R_{21}) + var(R_{20})}}{R_{20}} * 100$$

Where R_{20} is the ASDR for 2020 and R_{21} is the ASDR for 2021 for a given racial and ethnic group. These values were reported as percentages.

Variance for Rate Ratios:

© 2023 Lundberg DJ et al. *JAMA Network Open.*

The variance of the rate ratios was then calculated using the Delta method for calculating variance of ratios. This variance was then used to calculate the 95% confidence interval for the rate ratio.

$$var(RR) = \frac{var(R_i) * {R_r}^2 + var(R_r) * {R_i}^2}{{R_r}^4}$$

This variance was then used to calculate the 95% confidence interval for the rate ratios with the following formula:

$$\left(\frac{R_i}{R_r}\right) \pm 1.96 * \sqrt{var(RR)}$$

Where RR is the rate ratio, R_i is the ASDR being compared, and R_r is the referent group (non-Hispanic White) ASDR.

<u>Variance for Relative Changes in Rate Ratios:</u>
The variance of the relative change in rate ratio was calculated based on prior analysis by Chang et al. with the following formula:[3]

$$var\left(\frac{RR_{21} - RR_{20}}{RR_{20}}\right) = \left(\frac{RR_{21}}{RR_{20}}\right)^2 \left[\frac{var(RR_{21})}{{RR_{21}}^2} + \frac{var(RR_{20})}{{RR_{20}}^2}\right]$$

The 95% confidence interval was calculated with the following formula:

$$\left(\frac{RR_{21} - RR_{20}}{RR_{20}}\right) \pm 1.96 * \sqrt{var\left(\frac{RR_{21} - RR_{20}}{RR_{20}}\right)}$$

Where RR_{20} is the rate ratio for 2020 and RR_{21} is the rate ratio for 2021 for a given racial and ethnic group.

© 2023 Lundberg DJ et al. *JAMA Network Open.*

eAppendix 3. Decomposition Analysis Derivation and Formulas

Background:

A previously published decomposition analysis by Wrigley-Field et al. provided the conceptual and methodological basis for the decomposition analysis undertaken in this study.[4]

Our decomposition equation is adapted from techniques developed by Kitagawa (1955) and summarized in Preston, Heuveline, and Guillot (2001: 28-29).[5,6]

Assumptions:

The following analysis assumes that a hypothetical population in which all racial and ethnic groups were equally likely to live in large metros, medium/small metros, and nonmetro areas would have the same race or ethnicity-specific, geographic-specific mortality that the actual population does. The equivalent assumption is made for all standardized rates. For example, age-standardized mortality assumes that all racial and ethnic groups could have the same hypothetical age distribution even as they had their actual age-specific mortalities.

Objective:

We sought to decompose the change in racial disparities in age-standardized COVID-19 mortality between two time points into four components: (a) the geographically-standardized decrease in non-Hispanic Black or Hispanic mortality rates, (b) the geographically-standardized increase in non-Hispanic White mortality rates, (c) the mortality effect of movement in where deaths occurred from metropolitan to nonmetropolitan areas, and (d) the mortality effect of changes in the racial and ethnic population composition of metropolitan and nonmetropolitan areas. Here we present the decomposition equation and its derivation.

Terminologies:

We denote age-standardized mortality as a function of racial group *i=w,b* (denoting "Non-Hispanic White" and "Non-Hispanic Black" for concreteness, without loss of generality), time period *j=1,2* (representing distinct pandemic waves), and geography *k=u,t,s* (denoting large metropolitan, or "urban"; small/medium metropolitan, or "town"; and non-metropolitan, or "rural") as $m_{i,j,k}$. We denote the proportion of racial group *i* in geography *k* during period *j* as $c_{i,j,k}$; $\sum_k c_{i,j,k} = 1$. The mortality of racial group *i* in period *j*, aggregated over geographies, is $\bar{m}_{i,j} = \sum_k m_{i,j,k} c_{i,j,k}$.

To geographically standardize aggregate race and ethnicity-specific mortality rates, we use a standard geographic distribution, denoted c_k^{st} for each geography *k* (the standard distribution does not vary by race and ethnicity or year). The standard distribution we used to geographically standardize is the metro/non-metro distribution of the aggregate total of non-Hispanic Black, non-Hispanic White, and Hispanic populations averaged across the 5 pandemic waves from March 2020 through February 2022. For concision, we also introduce a parameter to reflect the difference between the actual composition of a racial and ethnic group during some period and this standardized composition: $\tilde{c}_{i,j,k} = c_{i,j,k} - c_k^{st}$.

© 2023 Lundberg DJ et al. *JAMA Network Open.*

Finally, for concision, we also introduce parameters to represent the change in geography-specific mortality for racial and ethnic group *i* between any two periods 1 and 2, $d_{i,k} = m_{i,2,k} - m_{i,1,k}$, and this change relativized to the change in urban mortality: $\tilde{d}_{i,k} = d_{i,k} - d_{i,u} = (m_{i,2,k} - m_{i,1,k}) - (m_{i,2,u} - m_{i,1,u})$.

Derivation:

The change between periods in racial and ethnic disparities in mortality is equivalent to the racial and ethnic disparity in the change in mortality:

$$(\bar{m}_{b,2} - \bar{m}_{w,2}) - (\bar{m}_{b,1} - \bar{m}_{w,1}) = (\bar{m}_{b,2} - \bar{m}_{b,1}) - (\bar{m}_{w,1} - \bar{m}_{w,1}) \tag{1}$$

Due to that equivalence, we proceed by geographically decomposing the changes in race and ethnicity-specific aggregate mortality ($\bar{m}_{i,2} - \bar{m}_{i,1}$) before plugging those components into Eq. (1) to produce our final decomposition equation.

The change in race and ethnicity-specific aggregate mortality can be geographically decomposed as:

$$\begin{aligned}\bar{m}_{i,2} - \bar{m}_{i,1} &= \sum_k \left[c_{i,1,k}(m_{i,2,k} - m_{i,1,k}) + (c_{i,2,k} - c_{i,1,k})m_{i,2,k}\right] \\ &= \sum_k \left[(c_k^{st} + \tilde{c}_{i,1,k})(m_{i,2,k} - m_{i,1,k}) + \left(c_k^{st} + \tilde{c}_{i,2,k} - (c_k^{st} + \tilde{c}_{i,1,k})\right) m_{i,2,k}\right] \\ &= \sum_k \left[c_k^{st}(m_{i,2,k} - m_{i,1,k}) + \tilde{c}_{i,1,k}(m_{i,2,k} - m_{i,1,k}) + (\tilde{c}_{i,2,k} - \tilde{c}_{i,1,k})m_{i,2,k}\right]\end{aligned} \tag{2}$$

Summing over all geography types k, Eq. 2 breaks the change in racial and ethnic group i's mortality between period 1 and period 2 into three terms. Eq. 2's first term, $\sum_k c_k^{st}(m_{i,2,k} - m_{i,1,k})$, gives a geographically-standardized change in mortality for racial or ethnic group k, in other words, the change in the mortality inequality that would have been observed strictly due to changes in racial or ethnic-specific mortality within each geography type in a hypothetical world in which both racial or ethnic groups had the same distribution across geographies as the standard population. Eq. 2's second term, $\sum_k \tilde{c}_{i,1,k}(m_{i,2,k} - m_{i,1,k})$, measures the effect of the racial and ethnic group's disproportionate representation in each geography *k* (weighted by the change in racial or ethnic-specific mortality in that geography), in other words, the change in the mortality inequality that was strictly due to the overrepresentation of the racial or ethnic group in geography types with large changes in mortality for that group. Eq. 2's third term, $\sum_k(\tilde{c}_{i,2,k} - \tilde{c}_{i,1,k})m_{i,2,k}$, measures the effect of changes between periods in the extent to which the population disproportionately resides in geography *k*. This third term is essentially trivial in this case ($\tilde{c}_{i,2,k} - \tilde{c}_{i,1,k}$ is close to zero).

To represent the extent to which disparities declined in response to mortality shifting away from large metropolitan areas, specifically, we further decompose Eq. 2's second term into specific geographies:

$$\begin{aligned}\sum_k \tilde{c}_{i,1,k}(m_{i,2,k} - m_{i,1,k}) &= \tilde{c}_{i,1,u}d_{i,u} + \tilde{c}_{i,1,t}d_{i,t} + \tilde{c}_{i,1,r}d_{i,r} \\ &= \tilde{c}_{i,1,u}d_{i,u} + \tilde{c}_{i,1,t}(d_{i,u} + \tilde{d}_{i,t}) + \tilde{c}_{i,1,r}(d_{i,u} + \tilde{d}_{i,r})\end{aligned}$$

© 2023 Lundberg DJ et al. *JAMA Network Open.*

$$= d_{i,u}(\tilde{c}_{i,1,u} + \tilde{c}_{i,1,t} + \tilde{c}_{i,1,r}) + \tilde{c}_{i,1,t}\tilde{d}_{i,t} + \tilde{c}_{i,1,r}\tilde{d}_{i,r}$$
$$= \tilde{c}_{i,1,t}\tilde{d}_{i,t} + \tilde{c}_{i,1,r}\tilde{d}_{i,r} \quad (3)$$

The simplification in the final line of Eq. 3 follows from each racial and ethnic group's disproportionate representation in each geography balancing out across geographies ($\tilde{c}_{i,1,u} + \tilde{c}_{i,1,t} + \tilde{c}_{i,1,r} = 0$). Thus, the portion of the overall change in racial and ethnic group *i*'s mortality ($\overline{m}_{i,2} - \overline{m}_{i,1}$) that reflects its disproportionate geographic distribution is represented in Eq. 3 as the extent to which racial and ethnic group *i*'s change in mortality between period 1 and period 2 was disproportionately concentrated in small/medium metropolitan and rural areas ($\tilde{d}_{i,t}$ and $\tilde{d}_{i,r}$, respectively), weighted by the extent to which racial and ethnic group *i* disproportionately resides in those respective geographies ($\tilde{c}_{i,1,t}$ and $\tilde{c}_{i,1,r}$).

Putting our expanded expression for the consequences of geographic shifts (Eq. 3) into the decomposition of the race and ethnicity-specific change in mortality in Eq. 2, we represent the total change in each racial group's COVID-19 mortality as:

$$\overline{m}_{i,2} - \overline{m}_{i,1} = \sum_k [c_k^{st}(m_{i,2,k} - m_{i,1,k})] + \tilde{c}_{i,1,t}\tilde{d}_{i,t} + \tilde{c}_{i,1,r}\tilde{d}_{i,r} + \sum_k [(\tilde{c}_{i,2,k} - \tilde{c}_{i,1,k})m_{i,2,k}] \quad (4)$$

Finally, since Eq. 4's terms combine additively, we can represent the contribution of each term to the change in racial and ethnic *disparities*, $(\overline{m}_{b,2} - \overline{m}_{w,2}) - (\overline{m}_{b,1} - \overline{m}_{w,1})$, as the difference between the value of each term for Non-Hispanic Black (or Hispanic) vs. Non-Hispanic White populations.

Thus, we decompose the change in racial and ethnic disparities between Period 1 and Period 2 into four distinct components:

a) Declining geographically-standardized non-Hispanic Black (or Hispanic) mortality: $\sum_k [c_k^{st}(m_{b,2,k} - m_{b,1,k})]$

b) Increasing geographically-standardized non-Hispanic White mortality: $\sum_k [c_k^{st}(m_{w,2,k} - m_{w,1,k})]$

c) The geographic shift in mortality from metropolitan to nonmetropolitan areas: $\tilde{c}_{b,1,t}\tilde{d}_{b,t} + \tilde{c}_{b,1,r}\tilde{d}_{b,r} - \tilde{c}_{w,1,t}\tilde{d}_{w,t} - \tilde{c}_{w,1,r}\tilde{d}_{w,r}$

d) Changes in the racial and ethnic population composition of metropolitan and nonmetropolitan areas: $\sum_k [(\tilde{c}_{i,2,k} - \tilde{c}_{i,1,k})m_{i,2,k}]$

© 2023 Lundberg DJ et al. *JAMA Network Open.*

eReferences

1. National Center for Health Statistics. *Vital Statistics of the United States: Mortality, 1999 Technical Appendix*.; 1999. https://wonder.cdc.gov/wonder/sci_data/mort/mcmort/type_txt/mcmort05/techap99.pdf

2. Truman BI, Chang MH, Moonesinghe R. Provisional COVID-19 Age-Adjusted Death Rates, by Race and Ethnicity - United States, 2020-2021. *MMWR Morb Mortal Wkly Rep*. 2022;71(17):601-605. doi:10.15585/mmwr.mm7117e2

3. Chang MH, Moonesinghe R, Athar HM, Truman BI. Trends in Disparity by Sex and Race/Ethnicity for the Leading Causes of Death in the United States-1999-2010. *J Public Health Manag Pract*. 2016;22 Suppl 1:S13-24. doi:10.1097/PHH.0000000000000267

4. Wrigley-Field E, Garcia S, Leider JP, Van Riper D. COVID-19 Mortality At The Neighborhood Level: Racial And Ethnic Inequalities Deepened In Minnesota In 2020. *Health Aff* . 2021;40(10):1644-1653. doi:10.1377/hlthaff.2021.00365

5. Kitagawa EM. Components of a Difference Between Two Rates*. J Am Stat Assoc. 1955;50(272):1168-1194. doi:10.1080/01621459.1955.10501299

6. Preston SH, Heuveline P, Guillot M. Demography: Measuring and Modeling Population Processes. Oxford: Blackwell Publishers Ltd; 2001.

© 2023 Lundberg DJ et al. *JAMA Network Open.*
