# Supplementary material for: COVID-19 Mortality by Race and Ethnicity in US Metropolitan and Nonmetropolitan Areas, March 2020 to February 2022
Source: JAMA Netw Open. 2023 May 2;6(5):e2311098. doi: 10.1001/jamanetworkopen.2023.11098 (PMC10155069; doi:10.1001/jamanetworkopen.2023.11098)
Supplement: Supplement 2. — Data Sharing Statement [file jamanetwopen-e2311098-s002.pdf]

Data Sharing Statement

Lundberg. COVID-19 Mortality by Race and Ethnicity in US Metropolitan and Nonmetropolitan Areas, March 2020 to February 2022. *JAMA Netw Open*. Published online May 2, 2023. doi:10.1001/jamanetworkopen.2023.11098

Data
Data available: No

Additional Information
Explanation for why data not available: COVID-19 mortality data used in the study are publicly available and can be accessed via the Centers for Disease Control and Prevention (CDC) WONDER online database. Population data used in the study are publicly available from the U.S. Census Bureau. Additional details about the data and programming code for replication can be accessed at the linked GitHub repository: https://github.com/The-Uncounted-Lab/covid-race-metro/.

© 2023 Lundberg DJ et al. *JAMA Network Open*.
